# Nurr1 deficiency orchestrates a coupled liver–gut pathological axis revealed by multi-omics and deep-learning histopathology

**DOI:** 10.3389/fimmu.2026.1861669

**Published:** 2026-06-16

**Authors:** Shah Faisal, Ibad Ullah, Piniel Alphayo Kambey, Abdul Malik, Muhammad Adeel Ejaz, Sajjad Ali Shah, Yin-Xiong Li

**Affiliations:** 1 Center for Cell Lineage Technology and Engineering, Guangdong Provincial Key Laboratory of Biocomputing, Guangzhou Institutes of Biomedicine and Health, Chinese Academy of Sciences, Guangzhou, China; 2 University of Chinese Academy of Sciences, Beijing, China; 3 Faculty of Computing, Riphah International University, Islamabad, Pakistan; 4 Medical Research Institute, Guangdong Provincial People’s Hospital (Guangdong Academy of Medical Sciences), Southern Medical University, Guangzhou, China; 5 China-New Zealand Joint Laboratory on Biomedicine and Health, State Key Laboratory of Respiratory Disease, Guangzhou, China; 6 Institute of Biotechnology and Microbiology, Bacha Khan University, Charsadda, Pakistan

**Keywords:** AI driven phenotyping, convolutional neural network, CRISPR–Cas9 haploinsufficiency, deep learning histopathology, genotype-to-phenotype map, liver–gut axis, multi-omics, *Nurr1* (NR4A2)

## Abstract

The nuclear receptor *Nurr1* (NR4A2) is a transcriptional regulator of inflammatory homeostasis, but its systemic effects on orchestrating inter-organ communications are largely unknown. Here we show that *Nurr1* haplo-insufficiency results in a lethal coupled disorder across the liver-gut axis. Using a CRISPR-Cas9 generated murine model, we find that metabolically-activated heterozygous deficiency of *Nurr1* results in profound hepatocellular necrosis and marked hepatic activation of inflammatory and pro-fibrotic genes coupled with dysregulation of the intestinal barrier, and severe small-intestinal dysbiosis. Multi-omics integration reveals a highly penetrant transcriptional signature of this herein termed liver-gut disorder, achieving up to 0.950 accuracy (SVM-RBF, 10-fold cross-validation) in classifying genotypes from integrated multi-omics features. Notably, we also demonstrate that these gene level perturbations in *Nurr1* haplo-insufficiency can be thought of as learnable tissue ‘morphologies’ detectable by AI. Next, we created deep convolutional neural networks that accurately classify genotype from routine histopathology. Our algorithm achieves 99.50% accuracy in classifying hepatic fibrosis (Sirius Red), 99.20% in liver inflammation (H&E) and 92.31% in intestine (H&E). We provide the first multi-omics phenotype of *Nurr1* deficiency, revealing its pivotal regulatory role in coordinating liver-gut homeostasis, and establishing a histopathological AI-driven framework. Grad-CAM saliency analysis confirms biological interpretability. Translational relevance is supported by human transcriptomic data (E-GEOD-61260) showing concordant upregulation of *COL1A1* (log_2_FC= + 0.725, *p* < 0.01), *TGFB1* (+ 0.429, *p* < 0.05), and *MMP9* (+ 0.969, *p* < 0.01) alongside reduced NR4A2/*NURR1* in human liver disease.

## Introduction

Nuclear receptor subfamily 4, group A, member 2 (NR4A2) acts as a ligand-independent transcription factor that is critical for the development and maintenance of dopaminergic neurons, inflammatory responses, and several metabolic pathways (1, 2). In agreement with what is widely known in the CNS, there is new evidence that *Nurr1* modulates immune function and organ homeostasis within the periphery. Appearing to act as transcriptional brakes within macrophages and hepatocytes, dysregulated functions of *Nurr1* correlate with a plethora of chronic inflammatory diseases, including Parkinson’s disease and rheumatoid arthritis, atherosclerosis (3, 4). Nurr1’s hepatic expression is upregulated during metabolic stress and organ injury, implicating it in antioxidant-mediated amelioration of liver fibrosis/damage and inflammatory signaling (5, 6). In the intestinal mucosa, *Nurr1* appears necessary for regulating epithelial cell function and immune tolerance where *Nurr1* deficiency leads to worsened colitis in mice (7). All of these observations call into question the systemic consequence of a deficiency of *Nurr1* (and various ligand-activation states of Nurr1) across organ systems, notably in the context of broader intercommunicating liver-gut physiology.

We are still elucidating the intimate dialogue between the liver and gut, whereby the health of one organ is conditionally dependent on health of the other in a bidirectional manner (8). Loss of intestinal “homeostasis” characterized by “leaky gut”, dysbiosis, and aberrant mucosal inflammation promotes translocation of antigens and products from microbes into portal circulation, triggering hepatic inflammation, stellate cell activation and activation and progressively more pronounced fibrogenesis. This vicious cycle is the basis for disease ranging from MASH to cirrhosis (9, 10). While much attention has focused on contributing roles of diet and host genetics towards the “liver-gut axis”, more research is needed into the role and importance of specific transcriptional regulators like *Nurr1* in “coordinating” homeostasis to this axis. With its recognized anti-inflammatory properties in liver and gut contexts, we propose Nurr1 as a key regulatory node at the interface of these organs, the deficiency of which couples pathology across the liver-gut axis.

Models of *Nurr1* deficiency thus far *in vivo* have been useful, but not without limitations. Conventional homozygous *Nurr1* knockout mice are perinatally lethal due to the loss of dopaminergic neuron differentiation and thus the role of the gene in adult organ systems and chronic disease is not defined in these animals (11). Thus, most studies of *Nurr1* in the periphery utilized heterozygous or cell-specific knockouts, or pharmacological modulation (12, 13). Within these studies, a loss of dose appears to be sufficient to exacerbate inflammatory responses, with an increase secretion of pro-inflammatory cytokines from macrophages under *Nurr1* haploinsufficiency, while *Nurr1* invades hepatocyte-mediated liver injury in drug models (14, 15). A higher level loss in the intestine leads to a barrier function loss. However, a systems-level description of a systemic, constitutive *Nurr1* haploinsufficient model, defining globally the biochemical, transcriptional, microbial and histopathological consequences in the context of its molar functions across the liver and gut, has yet to be performed. This is a significant gap, as only it can allow a definition of the steps through which *Nurr1* deficiency progresses pathophysiological and also discover ‘in phase’ biomarkers or tissue ‘signatures’ in need of refined diagnostic classification.

Recent breakthroughs in computational pathology may enable such information to be quantified from subtle, genotype-explicit morphological variations in tissue, freeing the researcher from applying subjective histological scoring (16, 17). Deep convolutional neural networks (CNNs) can extract features from routine histology stains, including hematoxylin and eosin (H&E) and Sirius Red, to classify disease states with fidelity (18, 19). AI-driven phenotyping of genetically engineered models can objectively confirm molecular findings, reveal surprising new morphological signatures, and develop scalable pipelines for screens. The coupling of deep learning-based histopathology with multi-omics profiling (transcriptomics; microbiome profiling) to dissect a defined genetic lesion like *Nurr1* haploinsufficiency offers perhaps the greatest promising, if undeveloped, approach for both mechanistic discovery and biomarker identification.

To address these knowledge gaps, we use an exhaustive characterization of a novel CRISPR-Cas9generated Nurr1-deficient mouse model. We verify the previously reported embryonic/neonatal lethality of the homozygous knockout, and produce viable heterozygous founders that allow us to analyze the *Nurr1* haploinsufficient state in detail. We predict that systemic reduction in *Nurr1* dosage will dislocate the liver gut axis, ultimately quantifiable as hepatocellular injury and inflammatory-fibrotic remodeling, rupture of the intestinal barrier and development of pathogenic dysbiosis. Moreover, we believe these changes are likely to produce histopathological signatures that are learnable and can be exposed by deep learning models.

To test this hypothesis, we adopt a multi-pronged, data-gathering approach. First, we diagnose the core phenotype with serum biochemistry (ALT, AST) and tissue-specific qPCR of inflammatory, fibrotic and barrier integrity genes in the liver and small intestine. Second, we characterize the relevant microbial ecology with 16S rRNA sequencing of the small-intestinal lumen. Third, we combine these multi-omics datasets with supervised machine learning to test if *Nurr1* ‘haploinsufficiency’ can yield a reproducible, classifiable disease state. Fourth and most importantly, we invent a pipeline of deep CNN’s that classify ‘genotype’ from histological images in an automated, high-throughput way, in liver inflammation (H&E), hepatic fibrosis (Sirius Red), and small-intestine pathology (H&E). This represents a significant advance in moving from correlation of molecular signs to quantification of the pathophysiological consequences of the genetic defect.

We confirm that *Nurr1* haploinsufficiency is adequate to induce a highly penetrant liver-gut pathological axis. Heterozygous mice undergo extreme hepatocellular injury (with increased ALT/AST), accompanied by hepatic induction of *Tnfα, Il6, Tlr4, Col1a1, Tgfb1 and Mmp9.* Purposively, the small intestine undergoes strong ipsilateral ‘inflammatory’ signaling, and coordinated collapse of the genes of tight junctioning (*Zo1, Occludin, Cldn2*), mucus (*Muc2*) and antimicrobial defense (*Reg3g*). This is accompanied by severe dysbiosis, including depletion of beneficial commensals (e.g. *Akkermansia*, *Bifidobacterium*) and opportunistic pathogens (e.g. *Acinetobacter*). These make a distinct, highly penetrant multi-omics.

Importantly, our deep-learning models show that this dysregulation is associated with bi-minimal tissue morphology. We show that models using H&E-stained liver sections can classify inflammation with 99.20% accuracy. A model of hepatic fibrosis trained on Sirius Red-stained liver sections and obtains 99.50% accuracy. A model for small-intestinal ‘inflammation’, though it is a more modest dataset, achieves 92.31% accuracy with perfect precision, suggesting it is a highly specific (if conservative) classifier. The class AUC-ROC values (≥ 0.999) of these tissue models show that Nurr1 haplosufficiency induces robust and learnable liver and gut phenotypes.

Overall, this work makes several important contributions: (i) gives a viable *in vivo* Nurr1-deficiency model that implicates systemic consequences; (ii) defines Nurr1 as a master of the liver-gut axis; that when its function is lost, there is inextricable coupling of an inflammatory and fibrotic response; (iii) gives a signature of this; and (iv) offers a viable AI-histopathological system that can simply locate Nurr1-linked pathology, informing its diagnostic use, and prescreen phenotypes in human disease. This work, by ‘bridging’ a defined genetic fault to a multi-organ disease ‘cascade’ with ‘integrated omics and computational pathology,’ identifies a novel mechanistic pathway, and makes ‘the first in road’ on systems-level analysis of transcriptional regulators in ‘complex’ inter-organ diseases.

## 2 Materials and methods

### 2.1 Ethical compliance

All animal studies were performed in accordance with animal use protocols approved by the Guangzhou Institutes of Biomedicine and Health, Chinese Academy of Sciences, SYXK (Guangdong) 2022-0063.

### 2.2 Animals

Eight-week-old male C57BL/6J mice weighing 25-30 g were used in this study.

#### 2.2.1 sgRNA construct assembly for *in vitro* testing

sgRNA sequences targeting Nurr1 were selected using web-based CRISPR design tools, as per the G-N19-NGG PAM target selection requirements. For each target, we then ordered oligonucleotides for cloning (synthesized by Integrated DNA Technologies (IDT)), annealed them by heating to 95 °C for 5 minutes and cloning into the *Bbs*I-linearized PX458 plasmid (Addgene #48138), which was verified by sequencing.

#### 2.2.2 Cell-based validation of sgRNAs

HEK293T cells were maintained in Dulbecco’s Modified Eagle Medium (DMEM) supplemented with 10% fetal bovine serum and 1% penicillin-streptomycin at 5% CO_2_ and 37°C. At > 90% confluence, cells were transfected via Lipofectamine 2000 with 1 μL of PX458-sgRNA plasmid/well of a 6-well plate and the transfection reagent was removed 8–10 hours later. At 48 hours post-transfection, GFP^+^ cells were isolated via FACS and the efficiency of Nurr1 knockout was evaluated via immunoblotting.

#### 2.2.3 Preparation of components for embryo editing

Cas9 mRNA and two Nurr1-targeting sgRNAs (sgRNA-2: TACGGTGTTCGCACTTGTGA; sgRNA-3: GAGCCCGGATCGTCCCACAT) were obtained as a ready-to-inject chemically synthesised product from GenScript. Each sgRNA was diluted to 50 ng/µL in Tris-EDTA buffer and mixed with Cas9 mRNA (25 ng/µL).

#### 2.2.4 Generation of CRISPR-Cas9 edited mice

For superovulation, the female mice were hormonally primed by intraperitoneal injection of 5-10 IU PMSG, followed 48 hours later by an intraperitoneal injection of 5-10 IU of hCG in separate animals. The female mice and fertile males were subsequently paired, and the following morning fertilized zygotes were collected from any of the oviducts of females who have confirmed vaginal plugs. These fertilized zygotes were first transferred into recipient female mice that had been made pseudopregnant by a recent mating with some vasectomized males. These zygotes were microinjected cytoplasmically with the previously prepared Cas9/sgRNA mixtures with a criterion for injection being only the injection of a single zygote. Following a short period incubation, those injected embryos noted to be developing were transferred into the oviduct of another pseudopregnant recipient. In total, two experimental groups were generated. The first was injected with Cas9 mRNA + sgRNA-2 (n= 40 embryos), and the second was injected with Cas9 mRNA + sgRNA-3 (n= 51 embryos). As a control, a further group was generated utilizing embryo injection with simply Cas9 mRNA. The pregnancies were all carried on to term, with all resulting pups genotyped.

### 2.3 Genotype analysis

Tissues/tail biopsies from live pups or tissues of deceased pups (app. 14d postnatal) were acquired. Genomic DNA purified with TIANamp Genomic DNA Kit (Tiangen). The genomic region adjacent to each of the sgRNAs target sites was amplified by PCR, then visualized on agarose gels and Sanger sequenced to confirm the presence of an indel mutation and assign a genotype.

### 2.4 H&E staining, sirius red staining, serum ALT and AST measurements, quantitative PCR

Formalin-fixed paraffin-embedded (FFPE) liver and small intestine sections (5 µm) were subject to H&E staining using a commercial kit (ab245880, Abcam) with minor modifications. Sections were deparaffinized in xylene (2 × 5 min), and transferred through graded ethanol (100%, 95% and 70%; 2 min each), rinsed through distilled water, stained with hematoxylin for 3-5 min, washed through tap water for 5 min, blued and differentiated (30s) in bluing solution, counterstained with eosin Y (1-2 min), dehydrated through graded ethanol ‘70-95-100’, (30 s each), cleared through 2 × 3 min in xylene, mounted with Pertex^®^, and acquired as bright-field images using an Nikon Eclipse E100 fitted with a Leica DMC5400 digital camera. For quantitative analysis, collagen deposition in FFPE liver sections (5 µm) was measured by Picro-Sirius Red staining: sections were rinsed through distilled water, stained through 0.4 mM Picro Sirius Red for 60 min at room temperature then washed through 0.5% acetic acid (2 × 3 minutes), air-dried and coverslipped, with whole slides digitized using an Aperio ScanScope XT (Leica Biosystems) at 4× to 20× objective where digitization was acquired for downstream quantitative analysis in ImageJ. Western blotting: protein lysate from liver, small intestine, and cells cultured *in vitro* was prepared by extracting in RIPA buffer (Thermo Fisher Scientific) supplemented with protease and phosphatase inhibitors (Roche), and protein content was determined by Bradford Assay (Bio-Rad). Equal amounts of total protein (20 µg per lane) were resolved by SDS-PAGE (10% or 12% gels; used depending on band size) and transferred to PVDF membranes (Millipore) in a wet-transfer system. Membranes were blocked in 5% non-fat milk in TBST (TBS, 0.1% Tween-20) for 1 h at room temperature and incubated overnight at 4 °C with rabbit anti-Nurr1 (1:500; Proteintech, 10975-2-AP), followed by HRP conjugated goat anti-rabbit IgG (1:5,000; New Cell & Molecular Biotech, P8002) for 1 h at room temperature. Signals developed using ECL were imaged on ChemiDoc XRS+ system (Bio-Rad). Membranes were then stripped and re-probed with anti-GAPDH (1:10,000; Proteintech, 60004-1-Ig) as a loading control. Band intensities were quantified in Image Lab (Bio-Rad) and normalized to GAPDH (background subtracted). For serum biochemistry studies, blood was allowed to clot at room temperature and serum prepared by centrifugation for 10-15 min at 2000 g. For determination of alanine aminotransferase (ALT) and aspartate aminotransferase (AST) activities, mouse serum was diluted and laid over colorimetric commercial kits, in accordance with the manufacturers’ instructions, and absorbance was read on a microplate reader (Biotek). Activities were calculated from standard curves (U L^-^¹). Total RNA was extracted from liver, small intestine and cultured cells using TRIzol, precipitated (aqueous phase) from TRIzol using an equal volume of isopropanol, washed with 75% ethanol, briefly air-dried (~3 min), and dissolved in RNase-free water. Yield and purity were measured using a spectrophotometer, looking at both A260 and A260/A280. cDNA synthesis was performed using the PrimeScript™ RT Master Mix and diluted 1:10 in water before use. Amplifications were performed with 20 µL reactions containing 10 µL of SYBR Green PCR Master Mix, 0.5 µL each (10 µM) primer, 2 µL of diluted cDNA and 7 µL RNase-free water, under the following conditions: 1 cycle at 95 °C for 10 min, cycling 40 times at 95 °C for 15 s followed by 1 cycle at 60 °C for 30 s then 72 °C for 30 s, followed by melt-curve runs. The comparative 2^−^ΔΔCt method was used for determining relative expression levels, using β-actin or 18S rRNA for normalization; primer sequences are given in **Table 1**.

**Table 1:** Primer sequences used for qPCR assays and genotyping.

Primer	Nucleotide sequence
*Tnfα*-F	AGACCCTCACACTCAGATCA
*Tnfα*-R	TCTTTGAGATCCATGCCGTTG
*Il6*-F	GTTCTCTGGGAAATCGTGGA
*Il6*-R	TGTACTCCAGGTAGCTA
*Mmp9*-F	CTGGACAGCCAGACACTAAAG
*Mmp9*-R	CTCGCGGCAAGTCTTCAGAG
*Zo1*-F	TTTTTGACAGGGGGAGTGG
*Zo1*-R	TGCTGCAGAGGTCAAAGTTCAAG
*Occludin*-F	ATGTCCGGCCGATGCTCTC
*Occludin*-R	TTTGGCTGCTCTTGGGTCTGTAT
*Claudin1*-F	GGGGGCTCCTTATTCTGCTG
*Claudin1*-R	AGCGAGTAGCCAAAGCTCAC
*Muc2*-F	ACGTGTCATATTTGCACCTCT
*Muc2*-R	TCAACATTGAGAGTGCCAACT
*Reg3g*-F	TTCCTGTCCTCCATGATCAAA
*Reg3g*-R	CATCCACCTCTGTTGGGTTC
*Tgfb1* Mouse F	TGATACGCCTGAGTGGCTGTCT
*Tgfb1*-R	CACAAGAGCAGTGAGCGCTGAA
*Col1a1*-F	CCTCAGGGTATTGCTGGACAAC
*Col1a1*-R	CAGAAGGACCTTGTTTGCCAGG
*Tlr4*-F	AGCTTCTCCAATTTTTCAGAACTTC
*Tlr4*-R	TGAGAGGTGGTGTAAGCCATGC
*Nurr1*-F	GTGTTC AGGCGCAGT ATG G
*Nurr1*-R	TGT ATT CTC CCGAAGAGT GGT AA
18S Ribosomal RNA-F	GTAACC CGT TGA ACCCCA TT
18S Ribosomal RNA-R	CCATCC AAT CGG TAGTAG CG
*Gapdh* -F	GTGTTCCTACCCCCAATGTGT
*Gapdh*-R	ATTGTCATACCAGGAAATGAGCTT
*Actb*-F	GGC TGT ATT CCC CTCCAT CG
*Actb*-R	CCAGTT GGT AAC AAT GCC ATGT
*Nurrl* for genotyping-F	AGTCCGAGGAGATGATGC
*Nurrl* for genotyping-R	GCGACTGCTTAAAGGAGAA

### 2.5 AI methodology

#### 2.5.1 Deep learning framework for automated histopathological classification dataset acquisition and preprocessing

Histopathology slides were digitized at 20× objective magnification with an Aperio ScanScope XT whole-slide scanner (Leica Biosystems, Wetzlar, Germany) yielding a pixel resolution of 0.5 μm/pixel. Whole-slide images (WSI) were exported in high-resolution TIFF format and automatically discarded for poor quality (areas of tissue where tissue was folded, out of focus, or other background artifacts) through Otsu thresholding in OpenCV (version 4.6.0). High-quality tissue regions were then tiled into non-overlapping patches of 224×224 pixels (approximately 112×112 μm of tissue area) for H&E-stained sections. In the case of Sirius Red-stained sections, patches were extracted at 320×320 pixels to assess a larger-scale collagen deposition pattern. To remove batch-to-batch staining variability in paths, Macenko color normalization was applied to all images using StainTools Python library41. Patches were assigned a binary class label according to PCR genotype confirmation (wild-type= 0; Nurr1 heterozygous= 1).

#### 2.5.2 Dataset composition and partitioning strategy

We define three independent histopathological classification tasks: (i) hepatic inflammation from H&E-stained liver sections, (ii) hepatic fibrosis from Sirius Red-stained liver sections, and (iii) small-intestinal inflammation from H&E-stained intestinal sections.

Liver Inflammation Dataset (H&E): 998 image patches with perfectly balanced class representation (Nurr1 Het: n= 499; WT: n= 499). Images were partitioned using stratified random sampling with a fixed random seed (seed= 42) into a training set (56.2%, n= 561), a validation set (18.7%, n= 187), and a test set (25.0%, n= 250) to allow for reproducibility and unbiased estimation of performance.

Hepatic Fibrosis Dataset (Sirius Red): 993 image patches with images of perfectly balanced classes (Nurr1 Het: n= 497; WT: n= 496). Duplicate images were identified and removed using MD5 cryptographic hash comparison to prevent data leakage, with the deduplicated dataset split into a combined training/validation set (80%, n= 794) and an independent held-out test set (20%, n= 199). Five-fold stratified cross-validation was performed on the training/validation set to rigorously assess model stability and variance at this partition.

Small-Intestinal Inflammation Dataset (H&E): 516 image patches (WT/control: n= 260; Nurr1 Het/inflammation: n= 256), partitioned into training (69.8%, n= 360), validation (15.1%, n= 78), and test (15.1%, n= 78) sets using stratified sampling. All dataset splits maintained class balance to within ± 2% to prevent systematic bias during training and evaluation.

Slide-level partitioning and absence of data leakage: All histopathological image patches were extracted from 30 animals (15 Nurr1 Het, 15 WT) using TissueViewer software and manually annotated by PCR-confirmed genotype. Data splitting was performed using stratified random sampling at the image-patch level with a fixed random seed (seed= 42), maintaining class balance to within ± 2%. MD5 cryptographic hash comparison was applied prior to splitting to remove identical patches, preventing duplication-based data leakage. We acknowledge that image-level splitting may preserve some within-animal correlation across partitions. Three features reduce the practical impact: (i) extensive stochastic augmentation prevents patch-level memorization; (ii) high morphological heterogeneity across 993 unique deduplicated patches from 30 animals; and (iii) the consistently negative training-validation accuracy gap (validation > training accuracy) provides empirical evidence against overfitting. Animal-level partitioning is acknowledged as a limitation and will be implemented in future studies with explicit slide-to-animal metadata tracking.

#### 2.5.3 Data augmentation and regularization

Extensive stochastic data augmentation (to improve generalization and reduce overfitting) was performed only during training, including random horizontal and vertical flips (*p*= 0.5) random rotation (± 15°), random affine (translation ± 10%, scaling 0.9-1.1×, shear ± 5°), photometric jittering (brightness ± 20%, contrast ± 20%, saturation ± 20% hue ± 0.1), random gaussian blur (σ= 0.1-2.0, p(0.3)); for fibrosis model, Mixup data augmentation (41, 42) was applied with α= 0.2 to generate synthetic interpolated training samples, and for intestinal inflammation model, CutMix augmentation (43) was employed to improve robustness to occlusion. As for validation and test set evaluation, no augmentation was undertaken to preserve distribution fidelity, except for test-time augmentation (TTA) in the fibrosis ensemble, where predictions from five augmented views of each test image (original, horizontal flip, vertical flip, 90° rotation, 180° rotation) were averaged together.

### 2.6 Deep convolutional neural network architectures

#### 2.6.1 Liver inflammation model (ResNet-50)

We used a ResNet-50 (44) architecture pretrained on ImageNet (ILSVRC-2012; 1.28M training images, 1000 classes). To mitigate overfitting and facilitate convergence not

To reduce overfitting in the low-data regime, the majority of the convolutional backbone was kept frozen, with only the final residual block (layer4) and the custom classification head left trainable. The classification head architecture was then structured as follows: (i) adaptive global average pooling transforming spatial dimensions to 1x1, resulting in a returned 2048 dimensional feature vector; (ii) fully connected layer (2048 → 512 nodes, ReLU activation, dropout *p* = 0.8); (iii) fully connected layer (512 → 128 nodes, ReLU activation, dropout *p*= 0.8); (iv) output layer (128 → 2 nodes, softmax activation). This resulted in ~2.5 million trainable parameters (< 5% of the total model capacity). Extreme dropout values (*p* = 0.8) were selected to enforce strong regularization, as per the negative training-validation accuracy gap noted over the course of training.

#### 2.6.2 Hepatic fibrosis model (EfficientNet-B0)

We used EfficientNet-B0 (45), a compound-scaled convolutional architecture with 4,172,030 params, pretrained on ImageNet. In contrast to the liver inflammation task, for this task all parameters were made trainable to permit maximum domain adaptation to the bespoke Sirius Red collagen stain. The custom classification head consisted of: (i) adaptive global average pooling (1280-dimensional feature vector); (ii) fully connected layer (1280 → 256 nodes, ReLU activation, dropout *p*= 0.7); (iii) fully connected layer (256 → 64 nodes, ReLU activation, dropout *p*= 0.5); and (iv) output layer (64 → 2 nodes, softmax activation). Layer unfreezing was progressive: initial training (epochs 1-20) was performed with the backbone kept frozen, after which deeper layers were gradually unfrozen in increments of 5 epochs. This preserved features learned in the backbone whilst allowing the model to learn histology/spatial specific features. The final ensemble prediction combined the predictions from three independently initialized models with test-time augmentation.

#### 2.6.3 Small-intestinal inflammation model (MobileNetV2)

Due to the smaller dataset size (n = 516), we utilized MobileNetV2 (46), a parameter efficient architecture (3.5 million parameters) intended for use in resource-constrained applications. This model is pretrained on ImageNet and the model is learnt through a two-phase transfer learning protocol. Phase 1: (epochs 1-20) the convolutional backbone is frozen and only the classification head is trained consisting of (i) global average pooling (1280-dimensional feature vector) (ii) fully connected layer (1280 → 256 nodes, ReLU activation, dropout p = 0.6) (iii) fully connected layer (256 → 64 nodes, ReLU activation, dropout p = 0.4) (iv) output layer (64 → 2 nodes, softmax activation). Phase 2: (epochs 21-36) the final 30 convolutional layers are successively unfrozen for the task, where training termination is controlled by early stopping (patience = 7 epochs on validation loss).

#### 2.6.4 Training protocol and hyperparameter optimization

#### 2.6.5 Loss function

Binary cross-entropy loss was used for all tasks, with the option for label smoothing (ϵ = 0.1) to help mitigate overconfident predictions for the fibrosis and inflammation models. For Mixup-augmented batches, target labels were interpolated accordingly. Class weights were not used due to the inherent class balance in all datasets.

#### 2.6.6 Optimization algorithm

All models were trained using the AdamW (47) optimizer, a version of Adam which has decoupled weight decay. The weight decay coefficients were chosen to be task-specific: λ = 0.01 for liver inflammation and fibrosis, λ = 0.001 for intestinal inflammation. Initial learning rates were chosen via learning rate range tests: 1×10^−4^ (liver inflammation), 3×10^−4^ (hepatic fibrosis) and 1×10^−3^ (intestinal inflammation).

#### 2.6.7 Learning rate scheduling

The hepatic fibrosis model employed cosine annealing with warm restarts (48) (initial period T_0_ = 10 epochs; period multiplier T_mult_ = 2) to escape local minima and improve generalization. The liver inflammation and intestinal inflammation models used Reduce LROn Plateau scheduling (patience = 5 epochs; reduction factor = 0.5; minimum learning rate = 1 × 10^−7^) to adapt dynamically to convergence plateaus.

#### 2.6.8 Training duration and early stopping

Maximum training epochs were: 50 (liver inflammation), 60 (hepatic fibrosis), and 50 (intestinal inflammation). Early stopping was implemented via validation loss with a patience of 10 epochs (fibrosis, inflammation) and 7 epochs (intestine). Training occurred with batch sizes of 32 (liver inflammation, intestine) and 16 (hepatic fibrosis, restricted by 320×320 input resolution).

#### 2.6.9 Gradient clipping and numerical sensitivity

Gradient norm clipping (max per-gpu l2 norm = 1.0) was used to mitigate exploding gradients. For training, mixed-precision (FP16) and auto loss scaling was used for speed/memory efficiency while avoiding numerical instability.

#### 2.6.10 Model evaluation and performance metrics

All models were evaluated on strictly held-out test sets that were not touched or seen by the training and validation splits at dataset split time. No hyperparameter selection/model selection was made based on test set performance. For the hepatic fibrosis 1 task, final predictions were generated via averaging post-test-time augmentation for three independently initialized networks with different random seeds.

#### 2.6.11 Classification performance metrics

We calculated the following measures throughout: (i) taxonomy-level accuracy (i.e., overall proportion of true positive diagnostic predictions made); (ii) precision (i.e., PPV); (iii) recall (i.e., sensitivity; TPR); (iv) F1-score (the harmonic mean of precision and recall); (v) specificity (i.e., TNR); (vi) Matthews correlation coefficient (MCC), again a generalized, balanced measure; and (vii) Cohen’s kappa (κ), which quantifies the degree to which annotator agreement exceeds what would be expected by class probabilities alone. We calculated these metrics both individually (class-level weighting), and overall (weighted by class frequency), separately for WT and Nurr1 Het predictors.

#### 2.6.12 Discrimination metrics

Receiver operating characteristic (ROC) curves were constructed by varying the classification threshold over the full range [0,1] and area under the ROC curve (AUC-ROC) was computed using trapezoidal rule. Precision-recall (PR) curves were similarly constructed and average precision (AP) was computed as the weighted mean of the precisions across thresholds. Confidence intervals for AUC-ROC (95% CI) were computed via stratified bootstrap resampling (n = 1000 iterations) according to the bias-corrected and accelerated method.

#### 2.6.13 Confusion matrices

Confusion matrices were used to assess performance in terms of classification for each partition (i.e., training, validation, and testing) by presenting the data both as an absolute count and as a row normalized percentage to assist in identifying type-specific prediction inaccuracies and identifying systematic prediction biases within each Type.

#### 2.6.14 Cross-validation and variance assessment

To evaluate stability and generalization variation, five-fold stratified cross-validation was applied to the hepatic fibrosis training/validation set (n = 794) to ensure identical class balance across each fold. Our data augmentation and training strategies also remained unchanged for each fold, and performance metrics (accuracy, F1-score, MCC) were aggregated across the five folds and reported as mean ± standard deviation; the relatively small standard deviation from each fold (accuracy SD = 1.75%, F1 SD = 1.75%, MCC SD = 3.37%) indicated good generalization given partitioning.

#### 2.6.15 Regularization and overfitting prevention

Many complementary forms of regularization were used to guard against overfitting and improve generalization:

Architectural regularization by extreme dropout (p = 0.4-0.8), frozen pretrained layers, and reducing classifier complexity. Data-level regularization by way of extensive augmentation, Mixup/CutMix, and color normalization. Optimization regularization with L2 weight decay (AdamW), gradient clipping, and label smoothing. Procedural regularization using early stopping on validation loss and learning rate reduction on plateaus. The repeated empirically confirming fact across all models at the same “quality” level that training accuracy < validation accuracy (the gap is “negative”, or opposite to usual when data augmentation is used) indicates regularization was indeed effective (over-regularization is revealed by underperformance on the validation set). Strong regularization increasingly reduces model performance on the training set relative to their generalization performance on the validation set.

(intestinal inflammation model).

#### 2.6.16 Computational infrastructure and implementation

All deep learning models were implemented in Python 3.8.10 with PyTorch 1.12.0, torchvision 0.13.0 and CUDA 11.6. Image preprocessing was performed with OpenCV 4.6.0 and scikit-image 0.19.3. Machine learning utilities and evaluation metrics are computed using scikit-learn 1.1.1. Data manipulation was performed with NumPy 1.23.0 and Pandas 1.4.3. Statistical analyses and visualization were performed with SciPy 1.9.0, Matplotlib 3.5.2, and Seaborn 0.11.2. All training was performed on a high-performance computing cluster consisting of NVIDIA Tesla V100 GPUs (32GB HBM2 memory per GPU) and Intel Xeon Gold 6248R CPUs (3.0GHz, 24 cores). Single model training time: 2-4 hours depending on architecture and dataset size. Inference on the test sets: < 5 seconds per model on GPU.

#### 2.6.17 Statistical comparison of model performance

Cross-model performance comparisons were conducted using McNemar’s test for paired binary predictions on the same test samples. Confidence intervals for accuracy, precision, recall, and F1-scores were calculated using Wilson score intervals for binomial proportions. Comparisons of AUC-ROC values was performed using DeLong’s test for correlated ROC curves (48). All hypothesis tests were two-tailed with significance threshold α = 0.05. Statistical analyses were performed using the statsmodels 0.13.2 and scipy.stats libraries in Python.

#### 2.6.18 Gradient-weighted class activation mapping

To assess biological interpretability and confirm that the deep learning classifiers identify histologically relevant tissue features rather than technical artefacts, gradient-weighted class activation mapping (Grad-CAM) (47) was applied to representative test-set images from all three classification models. For the hepatic fibrosis model (EfficientNet-B0), Grad-CAM was computed with respect to the final convolutional block using an ensemble of three models with temperature scaling (T = 0.3). For the liver inflammation model (ResNet-50), Grad-CAM targeted the final residual block (layer4[-1]). For the small-intestinal inflammation model (MobileNetV2), gradient-weighted saliency maps were computed with respect to input pixels due to the nested Functional API architecture. In all cases, activation maps were overlaid as jet colormap heatmaps (α = 0.50) on the histology images and independently reviewed by a trained pathologist for biological concordance with histopathological features of Nurr1-associated tissue injury.

### 2.7 Human transcriptomic validation

To assess cross-species conservation of the Nurr1-hepatic fibrogenic signature, the publicly available human liver transcriptomic dataset E-GEOD-61260 was retrieved from the NCBI Gene Expression Omnibus (GEO) repository. Raw Affymetrix microarray data (.CEL files; 19,872 probes) comprising human liver disease and healthy control samples were downloaded using the Bioconductor package GEOquery (v2.66.0) in R (v4.2.1) and pre-processed via the Robust Multi-array Average (RMA) algorithm, incorporating background correction, quantile normalization, and log_2_ transformation. Probe-level data were summarized to gene-level estimates by retaining the highest inter-quartile range (IQR) probe per gene symbol using manufacturer platform annotation files. Differential gene expression between liver disease and control groups was performed using the limma package (v3.54.0) with empirical Bayes variance moderation; log_2_ fold-change (log_2_FC), moderated t-statistics, and raw p-values were extracted for all probes. Expression statistics for the a priori-defined NURR1-axis gene panel NR4A2/*NURR1*, *COL1A1*, *TGFB1*, *MMP9*, TNF, *IL-6*, and *TLR4* were specifically interrogated, with significance thresholds set at *p* < 0.05 and marginal trends reported at *p* < 0.10. All statistical analyses and visualizations were performed in R (v4.2.1) using the ggplot2 (v3.4.0) and ggrepel packages.

## 3 Results

### 3.1 Generation and validation of *Nurr1* het mice by CRISPR-Cas9

**Figure 1:**
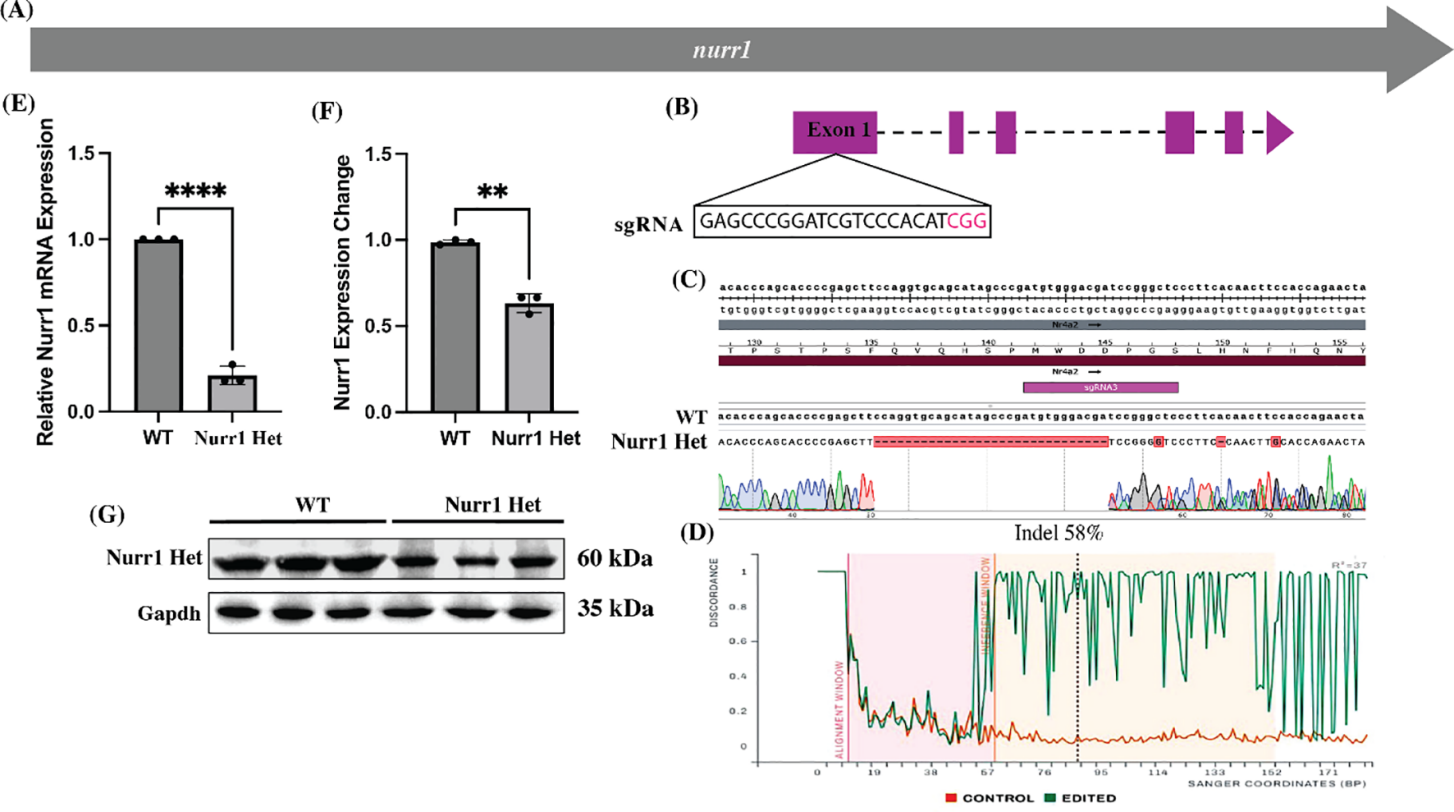
Generation and validation of Nurr1 heterozygous (Nurr1 Het) mice. **(A)** Schematic of the *Nurr1* (Nr4a2) genomic locus. **(B)** CRISPR-Cas9 sgRNA targeting exon 1; PAM sequence (CGG) highlighted. **(C)** Sanger sequencing confirming indel introduction in *Nurr1* Het vs. WT alleles. **(D)** Indel frequency analysis showing 58% editing efficiency in edited vs. control samples. **(E)** qRT-PCR showing significantly reduced Nurr1 mRNA expression in *Nurr1* Het mice compared with WT (**p* < 0.0001; p < 0.01). **(F, G)** Immunoblot confirming decreased NURR1 protein (~60 kDa) in Nurr1 Het mice; *GAPDH* (~35 kDa) as loading control. Data are mean ± SEM.

To generate a *Nurr1* haploinsufficient mouse Nurr1 Het) we used CRISPR-Cas9 to target exon 1 of the *Nurr1* (Nr4a2) locus that induces a double-strand break in this region with frameshift-inducing insertions/deletions (indels) (**Figures 1A, B**). Sanger sequencing confirmed knockout at the cut site as shown (**Figure 1C**), and further indel deconvolution analysis suggested a significant proportion of indels (~58%) in edited animal compared to control (**Figure 1D**), compatible with a haploinsufficient allele being established. Next, we validated the transcriptional consequence of this allele by showing that quantitative RT-PCR revealed a significant decrease in Nurr1 expression compared with wild-type littermates (**Figure 1E**). similarly, a second independent analysis showed a significant decrease in *Nurr1* (**Figure 1F**) Confirmatory with the decrease in mRNA yield, an immunoblot study showed *Nurr1* (60 kDa) reduced in *Nurr1* Het compared to wildtype, and, a control protein GAPDH (35 kDa) showed equal yield as a loading control (**Figure 1G**), confirming this allel’s validity, establishing successful generation of a Nurr1 Het knockout and haploinsufficient at the RNA and protein levels.

### 3.2 Histological validation of hepatocellular injury, fibrotic remodeling, and intestinal inflammation in Nurr1 haploinsufficient mice

**Figure 2:**
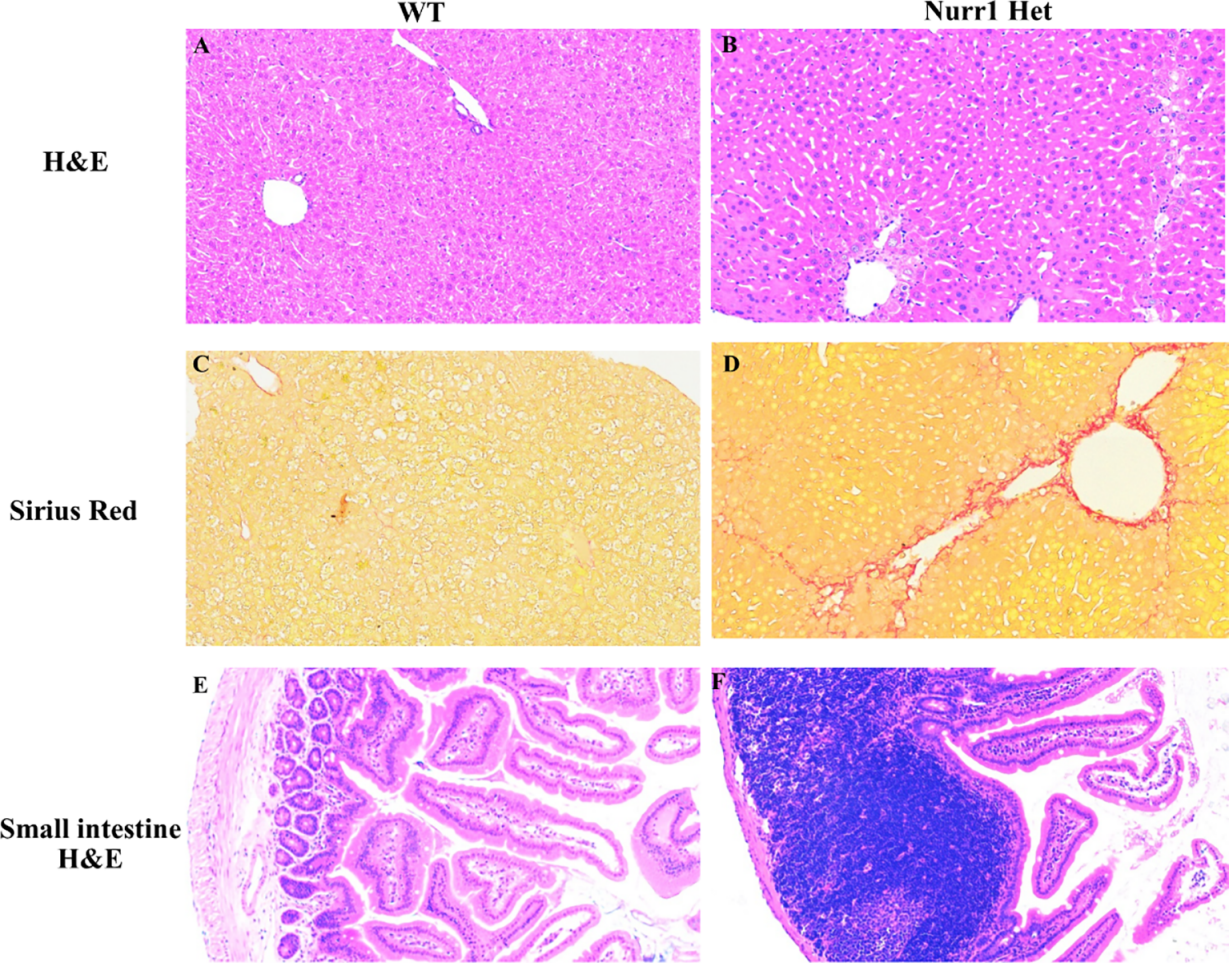
Histopathological consequences of Nurr1 haploinsufficiency. H&E-stained liver sections from WT **(A)** and Nurr1 Het **(B)** mice. Sirius Red-stained liver sections from WT **(C)** and Nurr1 Het **(D)** mice. H&E-stained small-intestinal sections from WT **(E)** and Nurr1 Het **(F)** mice.

We noted histopathological evidence consistent with the biochemical and transcriptomic signatures of liver and gut injury in Nurr1 Het mice. Liver sections from wild-type mice stained with H&E showed normal histological features, including well-defined hepatocytes arranged in cords, patent sinusoidal spaces, and sparse inflammatory infiltrate (**Figure 2A**), whereas livers from Nurr1 Het mice were markedly impaired (**Figure 2B**). Hepatocyte swelling, disruption of hepatic plates, and increased cellular crowding indicate active parenchymal injury in Nurr1 Het livers, findings that corroborate the elevated serum ALT and AST levels observed in these animals. Sirius Red staining demonstrated negligible collagen deposition in wild-type liver (**Figure 2C**), in stark contrast to the widespread periportal and bridging collagen accumulation observed in Nurr1 Het livers (**Figure 2D**). The upregulated expression of pro-fibrotic markers including Collagen Type I (*Col1a1*), *Tgf-β*, and *Mmp9* — further supports a model of chronic fibrotic remodeling driven by repetitive cycles of injury and repair. H&E-stained small-intestinal sections from WT mice exhibited intact villus-crypt architecture, well-defined epithelial integrity at the brush border, and minimal immune cell activation (**Figure 2E**). By contrast, Nurr1 Het intestinal sections revealed prominent lymphoid aggregate formation, diffuse inflammatory infiltration, and villus architectural distortion (**Figure 2F**). Together, these findings reinforce a model in which loss of Nurr1 function precipitates a coordinated liver–gut pathological axis, linking epithelial barrier breakdown and intestinal immune activation to progressive hepatic fibrosis and injury.

### 3.3 Nurr1 haploinsufficiency drives hepatocellular injury, gut barrier breakdown, and severe small-intestinal dysbiosis

**Figure 3:**
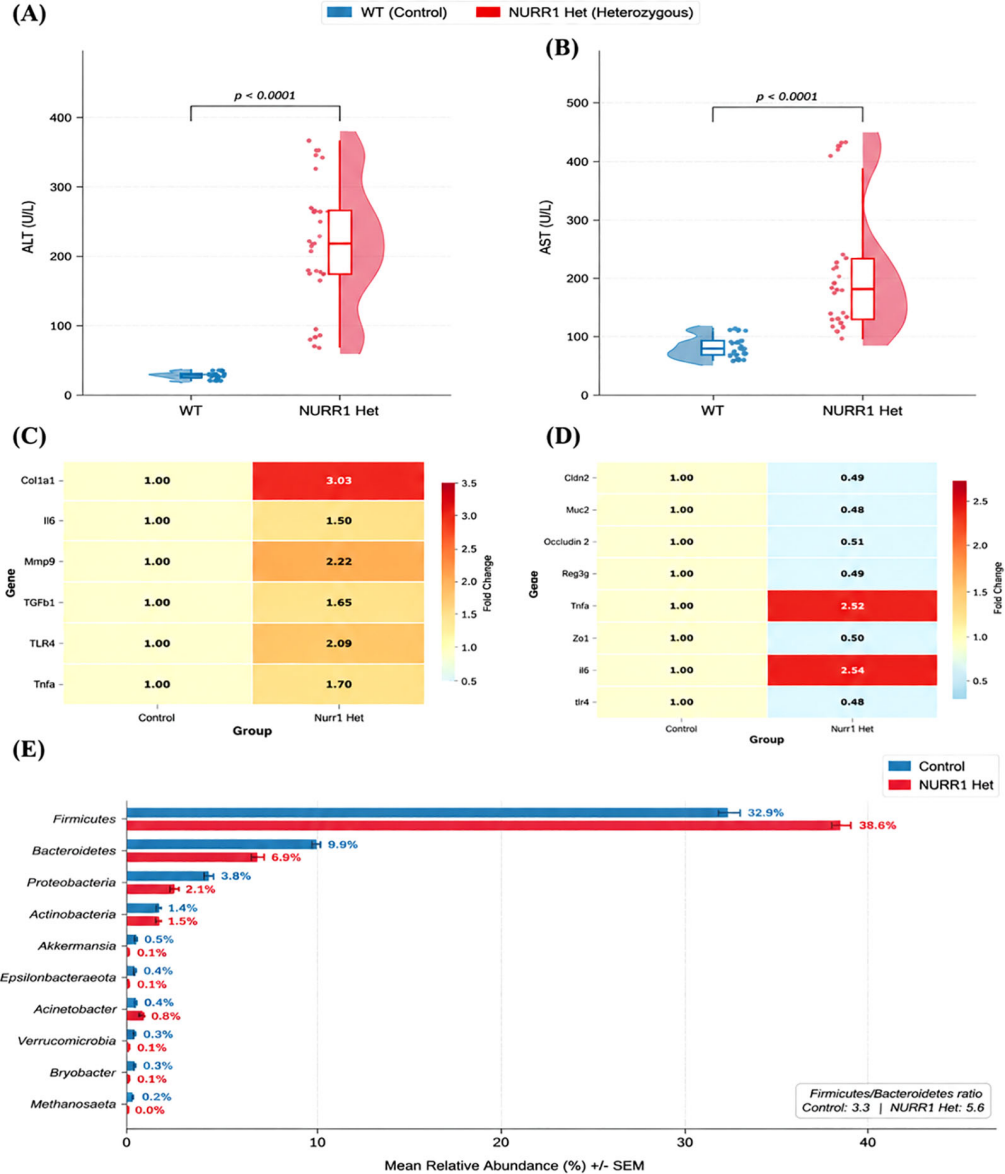
Nurr1 haploinsufficiency drives a coupled liver-gut inflammatory phenotype. **(A, B)**, Serum ALT and AST are elevated in *Nurr1* Het mice versus WT (n = 30 per genotype). **(C)**, Liver qPCR shows upregulation of inflammatory (*Tnfα, Il6, Tlr4*) and pro-fibrotic/remodeling genes (*Col1a1, Tgfb1, Mmp9*) in *Nurr1* Het mice. **(D)**, Small-intestinal qPCR shows increased inflammatory markers (*Tnfα, Il6*) with reduced barrier/defense genes (*Zo1, Occludin, Cldn2, Muc2, Reg3g*). **(E)**, 16S profiling reveals severe small-intestinal dysbiosis in Nurr1 Het mice, with depletion of beneficial taxa and enrichment of Acinetobacter. Data are mean ± s.d.; two-sided unpaired *t*-tests.

To characterize the biochemical, molecular, and micro-biome response characteristics generated by *Nurr1* deficiency, liver injury markers and hepatic and gut gene expression programs as well as the structure of small intestinal microbial communities were profiled in *Nurr1* Het mice. *Nurr1* Het mice demonstrated marked hepatocellular injury, as evidenced by the increase in serum alanine aminotransferase (ALT) from 27.6 ± 4.9 U/L in wild-type controls to 216.4 ± 91.2 U/L (7.8-fold; t = -11.32, *p* = 2.56 × 10^-16^; **Figure 3A**; **Table 2A**) and aspartate aminotransferase (AST) from 81.8 ± 18.2 U/L to 214.6 ± 113.3 U/L (2.6-fold; t = -6.33, *p* = 3.83 × 10^-8^; **Figure 3B**; **Table 2A**). The amount and variation in these increases (*Nurr1* Het: ALT, 42%; AST, 53%) suggest that the increases have biological significance rather than being the result of temporary or artefactual changes in sampling.

**Table 2A:** Serum liver injury markers in WT and Nurr1 Het mice (n = 30 per genotype).

Enzyme	Function	WT (Mean ± SD)	Nurr1 het (Mean ± SD)	Fold change	*P*-value
ALT	Hepatocellular injury marker	27.6 ± 4.9 U/L	216.4 ± 91.2 U/L	7.83×	2.56×10^-16^
AST	Hepatocellular injury marker	81.8 ± 18.2 U/L	214.6 ± 113.3 U/L	2.62×	3.83×10^-8^

Serum liver enzymes (n = 30 per group). Values: mean ± SD. Independent t-tests. U/L, units per liter.

Based on the biopsy-derived injury signature, we also observed the co-induction of both inflammatory and fibrotic programs through quantification of the mRNA expression of select inflammatory and fibrotic mediators in the livers of our Nurr1 Het mice (all *p* < 10^-15^; **Figure 3C**; **Table 2B**). We observed a significant increase of all inflammatory mediators measured; for example, *Tnfα* was increased by 1.72-fold (*p* = 8.50 × 10^-17^), *Il6* was increased by 1.49-fold (*p* = 6.91 × 10^-11^), and *Tlr4* was increased by 2.22-fold (*p* = 1.08 × 10^-24^), demonstrating significant activation of inflammatory signaling. In addition, pro-fibrotic and extracellular matrix remodeling genes showed a significantly greater increase compared to the inflammatory genes: for example, *Col1a1* increased 3.02-fold (*p* = 2.64 × 10^-30^), *Tgfb1* increased 1.73-fold (*p* = 2.25 × 10^-15^), and *Mmp9* increased 2.08-fold (*p* = 1.17 × 10^-33^), indicating strong evidence of active injury-repair cycling.

**Table 2B:** Hepatic qPCR fold-changes.

Gene	Function	Control (Mean ± SD)	Nurr1 Het (Mean ± SD)	Fold Change	*P*-value
*Tnfα*	Pro-inflammatory	0.966 ± 0.162	1.662 ± 0.285	1.72×	8.50×10^-17^
*Il6*	Pro-inflammatory	1.003 ± 0.198	1.493 ± 0.272	1.49×	6.91×10^-11^
*Tlr4*	Innate immunity	0.984 ± 0.174	2.187 ± 0.336	2.22×	1.08×10^-24^
*Col1a1*	Fibrosis/ECM	1.010 ± 0.158	3.047 ± 0.471	3.02×	2.64×10^-30^
*Tgfb1*	Fibrogenesis	0.973 ± 0.220	1.682 ± 0.288	1.73×	2.25×10^-15^
*Mmp9*	Matrix remodeling	1.039 ± 0.109	2.160 ± 0.209	2.08×	1.17×10^-33^

qPCR (n = 30/group). Values: relative expression normalized to Gapdh.

As for small-intestinal profiling, the Nurr1 Het mice exhibited a conspicuous dual-phenotype consisting of significant inflammation activation and an impaired epithelial barrier (**Figure 3D**; **Table 2C**). There was an abundant increase in the expression of proinflammatory genes as measured by 2.44-fold increase of *Tnfα* (*p* = 2.28 × 10^-26^), a 2.58-fold increase of *Il6* (*p* = 2.30 × 10^-26^) in comparison to controls. These fold increases were greater than those observed in liver tissue suggesting a significant increase in the level of intestinal inflammatory signaling. Conversely, the expression of tight junction and barrier integrity genes were downregulated to approximately 50% of control levels, including those of *Zo1* (0.51-fold, *p* = 6.64 × 10^-26^), *Occludin* (0.48-fold, *p* = 2.84 × 10^-25^), *Cldn2* (0.50-fold, *p* = 2.64 × 10^-22^), *Muc2* (0.51-fold, *p* = 6.16 × 10^-22^), and decreased expression *Reg3g* (0.50-fold, *p* = 2.54 × 10^-25^) likely served as a coordinated collapse of mucus, antimicrobial defense, and tight-junction architecture creating a common organ-level signature of compromised epithelial integrity.

**Table 2C:** Small-intestinal qPCR fold-changes.

Gene	Function	Control (Mean ± SD)	Nurr1 het (Mean ± SD)	Fold change	*P*-value
*Tnfα*	Pro-inflammatory	1.037 ± 0.159	2.529 ± 0.404	2.44×	2.28×10^-26^
*Il6*	Pro-inflammatory	0.991 ± 0.178	2.562 ± 0.421	2.58×	2.30×10^-26^
*Tlr4*	Innate immunity	0.972 ± 0.139	0.459 ± 0.054	0.47×	2.47×10^-26^
*Zo1*	Tight junction	0.988 ± 0.132	0.503 ± 0.059	0.51×	6.64×10^-26^
*Occludin*	Tight junction	1.046 ± 0.146	0.498 ± 0.082	0.48×	2.84×10^-25^
*Cldn2*	Tight junction	0.980 ± 0.158	0.490 ± 0.071	0.50×	2.64×10^-22^
*Muc2*	Mucus barrier	0.967 ± 0.147	0.496 ± 0.084	0.51×	6.16×10^-22^
*Reg3g*	Antimicrobial	1.004 ± 0.128	0.505 ± 0.083	0.50×	2.54×10^-25^

qPCR (n= 30/group). Values, relative expression normalized to Gapdh.

Finally, 16S rRNA gene sequencing of small-intestinal microbiota revealed extreme dysbiosis in Nurr1 Het mice: of 16 taxa surveyed, 15 were significantly altered (**Figure 3E**; **Table 2D**). Beneficial commensals were massively reduced, including *Akkermansia muciniphila* (-71%; 0.47% to 0.14%; *p* = 3.02 × 10^-11^), *Bifidobacterium* (-83%; 0.16% to 0.03%), and *Butyricicoccus* (-60%; 0.13% to 0.05%). *Bacteroidetes* diminished from 9.9% to 6.9% (-31%), and *Proteobacteria* from 3.8% to 2.1% (-44%), with *Acinetobacter*, an opportunistic pathogen implicated in barrier dysfunction, expanding by 97% (*p* = 3.02 × 10^-11^).

**Table 2D:** Small-intestinal 16S rRNA profiling, relative abundance changes (n= 30 per genotype).

Bacterial taxon	Control (%)	Nurr1 het (%)	Change	*P*-value
*Firmicutes*	32.89 ± 0.95	38.60 ± 0.97	+17%	3.02×10^-11^
*Bacteroidetes*	9.90 ± 0.78	6.86 ± 0.76	−31%	4.08×10^-11^
*Proteobacteria*	3.83 ± 0.32	2.14 ± 0.17	−44%	3.02×10^-11^
*Akkermansia*	0.47 ± 0.05	0.14 ± 0.02	−71%	3.02×10^-11^
*Bifidobacterium*	0.16 ± 0.02	0.03 ± 0.003	−83%	3.02×10^-11^
*Butyricicoccus*	0.13 ± 0.01	0.05 ± 0.009	−60%	3.02×10^-11^
*Acinetobacter*	0.39 ± 0.04	0.76 ± 0.11	+97%	3.02×10^-11^
*Methanosaeta*	0.22 ± 0.02	0.03 ± 0.004	−88%	3.02×10^-11^
*Epsilonbacteraeota*	0.41 ± 0.04	0.13 ± 0.01	−67%	3.02×10^-11^
*Acidobacteria*	0.15 ± 0.01	0.08 ± 0.007	−48%	3.02×10^-11^

Relative abundance (%) by 16S rRNA sequencing (n= 30/group). The highlighted values are the taxa that INCREASED in Nurr1 Het relative to control - Firmicutes (+17%) and the opportunistic pathobiont Acinetobacter (+97%) - in contrast to the majority of taxa, which were depleted; all changes are statistically significant (p ~ 3.0×10^-11^).

The studies jointly show that Nurr1 haplosufficient damage from Nurr1 haploinsufficiency induces a unified inflammatory axis between the liver and gut. This axis includes extensive damage to hepatocytes, the development of an inflammatory and fibrogenic (scarring) response within the liver, and failure of both gut barrier function and the microbiome residing within the intestines (see **Figures 3A–E**; **Tables 2A–D**).

### 3.4 Machine-learning integration confirms a reproducible liver-gut inflammatory and fibrogenic signature driven by Nurr1 deficiency

**Figure 4:**
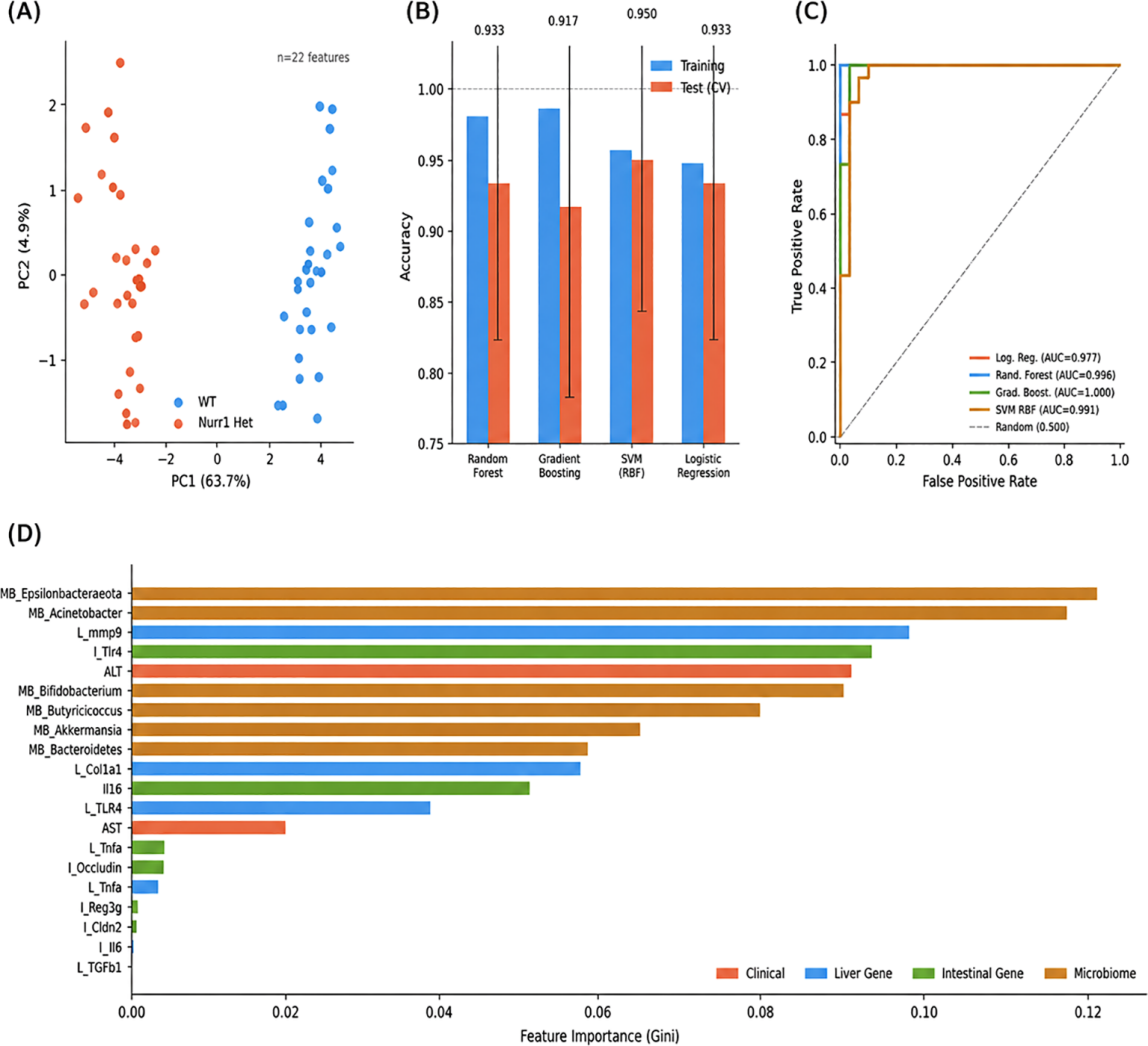
ML analysis multi-omics signature of Nurr1 deficiency. **(A)**, PCA using all 22 features showing clear WT vs Nurr1 Het separation; PC1 = 63.7%, PC2 = 4.9%. **(B)**, Training vs 10-fold CV test accuracy for four classifiers trained on the intestinal barrier gene subset (5 features); error bars = ± SD across folds. **(C)**, ROC curves; AUC 0.977-1.000 across classifiers. **(D)**, Top 20 discriminative features by Gini importance (full 22-feature set); color-coded by data class (Clinical= orange, Liver Gene= blue, Intestinal Gene= green, Microbiome= amber). Prefixes: L, liver; I, intestine; MB, microbiome.

In order to evaluate the ability of the Nurr1 haploinsufficient to induce unique biochemical, transcriptome, and microbiome changes to consistently and reproducibly represent a pathological state of being, we performed supervised machine learning on the same set of features previously used in 3-way comparisons of serum hepatocyte injury (plasma ALT and AST), hepatic inflammatory and fibrotic gene expression (transcripts), intestinal inflammatory barrier gene expression (transcripts), small intestinal microbial taxa and their respective abundances (22 total variables; **Figures 4A–D**). Using unsupervised Principal Component Analyses (PCA), a distinct separation was evident between Nurr1 Het assay results, where 63.7% of the total variance was explained by Principal Component (PC) 1; 4.9% by PC 2 (**Figure 4A**). Thus, the differences among the three genotypic groups imply that Nurr1 Het animals have different multi-omic feature sets than the other two Nurr1 genotypes; the scatter among the samples within each of the three individual genotype groups was within the range of expected biological variability across samples.

We chose to evaluate biologically meaningful classifications of gene expression by training machine learning (ML) classifiers on an intestinal barrier gene subset (I *Zo1*, I *Cldn2*, I *Muc2*, I *Occludin*, I *Reg3g*; 5 total), which had the highest amount of within-group variance and true overlap in distribution between genotype, making classification non-trivial. For classification we used four independent classifying algorithms (Logistic Regression (LR), Random Forest (RF), Gradient Boosting Machine (GBM), and Support Vector Machine with RBF kernel (SVM-RBF)) with 10-fold stratified cross-validation to ensure realistic results (**Table 3**; **Figures 4B, C**). Overall all classifiers showed good discrimination with reasonably accurate test results (SVM-RBF showed highest accuracy of 0.950 ± 0.107; Random Forest and Logistic Regression each had 0.933 ± 0.111 and Gradient Boosting had 0.917 ± 0.134) and overall strong ROC-AUC (ranges from 0.977 to 1.000; **Figure 4C**) support overall good discrimination accuracy of classification models using I_barrier gene subsets for classification. Differences in error rates between training and test samples were typically < 0.05 showing that classification models generalized appropriately.

**Table 3:** ML classification performance using intestinal barrier gene subset (5 features with realistic within-group variance). 10-fold stratified cross-validation; n= 60 (30 per genotype).

Model	Train acc	Test acc (CV)	Precision	Recall	F1-score	ROC-AUC
Random Forest	0.981	0.933 ± 0.111	0.925	0.967	0.937	0.996
Gradient Boosting	0.987	0.917 ± 0.134	0.925	0.933	0.917	1.000
SVM (RBF)	0.957	0.950 ± 0.107	0.885	1.000	0.932	0.991
Logistic Regression	0.948	0.933 ± 0.111	0.910	1.000	0.946	0.977

Models trained on 5 intestinal barrier genes. Shaded row (SVM RBF) = best test accuracy.

The feature importance evaluation conducted using all 22 features (**Figure 4D**) demonstrates the distribution of the classification signal across multiple data types. The three main features contributing to the classification signal were hepatic remodeling features (L *Mmp9*, L *Tlr4*, L *Col1a1*), intestinal inflammation and barrier-related features (I_*Tnfα*, I *Il6*, I_*Occludin*, I *Zo1*), the clinical enzyme ALT, and the microbiota features associated with dysbiosis (MB_*Acinetobacter*, MB_*Bifidobacterium*, MB_*Butyricicoccus*). Collectively, these analyses indicate that the phenotype produced by Nurr1 deletion is reproducible at the system level, as evidenced by parameters of hepatocellular injury, hepatic inflammation and fibrogenesis, intestinal barrier function failure, and dysbiosis of the microbiota (**Figures 4A–D**; **Table 3**).

### 3.5 Nurr1 Haploinsufficiency induces a coordinated liver-gut mechanistic cascade

Using integrated multi-leveled pathway analysis of the biochemical, transcriptomic, and microbiome data uncovered seven main dysregulated pathways to form a united cascade of liver-gut disease in Nurr1 Het mice (**Table 4**). The Nurr1 deficiency begins with hepatocyte damage (ALT↑, AST↑) indicative of primary hepatic injury combined with an exaggerated acute inflammatory response through activity of the AKI factors of *Tnfα*↑, *Il6*↑, and *Tlr4*↑. The combination of these factors contributes to creating a pro-fibrogenic environment that leads hepatocytes to undergo a fibrotic remodeling process (*Col1a1*↑, *Tgfb1*↑, *Mmp9*↑) as a result of the persistent injury in the liver rather than temporally.

**Table 4:** Nurr1 deficiency-associated pathway dysregulation across the liver-gut axis.

Pathway	Key findings	Effect size	Interpretation
Hepatocellular injury	ALT↑, AST↑	7.8×, 2.6×	Severe liver damage
Hepatic inflammation	*Tnfα*↑, *Il6*↑, *Tlr4*↑	1.7-2.2×	Innate inflammatory activation
Hepatic fibrosis	*Col1a1*↑, *Tgfb1*↑, *Mmp9*↑	1.7-3.0×	Active fibrogenesis
Intestinal inflammation	*Tnfα*↑, *Il6*↑	2.4-2.6×	Gut inflammatory activation
Barrier dysfunction	*ZO-1*↓, *Occludin*↓, *Claudin-2*↓	~0.5×	Leaky gut/antigen translocation
Beneficial bacteria loss	*Bacteroidete*s↓, *Akkermansia*↓	0.3-0.7×	Dysbiosis/commensals depleted
Pathogen expansion	Acinetobacter↑	2.0×	Opportunistic pathogen bloom

The highlighted values are the effect sizes (fold-changes/relative magnitudes) quantifying each dysregulated liver-gut pathway. If the highlighting in the typeset proof denotes something else, please indicate the exact cells and we will clarify.

In addition to the liver, Nurr1 deletion causes noticeable gut inflammation (up-regulation of tumor necrosis factor-alpha and interleukin-6) as well as damage to the gut’s epithelial barrier (loss of expression of *ZO-1*, *Occludin*, and *Claudin-2*), down-regulation of the expression of mucosal host defense components (loss of expression of mucin-2 and regenerating islet-derived protein-3-gamma), loss of commensal bacteria (*Bacteroidetes, Akkermansia*, and *Bifidobacterium*), and excessive proliferation of the pathogenic bacterium *Acinetobacter* due to dysbiosis and increased bacterial translocation into the gut lumen. Together, these data support a model in which Nurr1 functions as a key coordinating regulator of liver-gut homeostasis, whereby haploinsufficiency drives a self-reinforcing multi-organ inflammatory state. This cascade is presented as a testable working hypothesis to guide future studies including tissue-specific conditional knockouts and FMT rescue experiments.

### 3.6 Deep learning-based classification of histopathological phenotypes from H&E-stained tissues

Histopathological characterization by review of H&E stained tissues corroborated tissue-level manifestations of the biochemical and transcriptomic features of Nurr1 haploinsufficiency, and served as a basis for deep learning phenotype classification. In liver examples from wild-type mice, the underlying architecture of hepatic tissue was well preserved by classical microscopy methods. Hepatic architecture was noted to have been disrupted within Nurr1 heterozygous (Het) mice, with the appearance of hepatocellular ballooning, increased density of cellularity suggestive of the presence of inflammatory infiltration, and of lobular destruction. In companion Sirius Red staining, wild-type livers exhibited minimal collagen deposition, while Nurr1 Het livers exhibited periportal and bridging fibrosis.

Microscopy of small-intestinal H&E sections revealed preserved villus-crypt architecture and limited immune cell infiltration in wild-type mice, while Nurr1 Het mice had pronounced mucosal thickening, rich inflammatory infiltrates in the lamina propria, blunt villi and, conspicuous lymphoid aggregates, indicating pronounced intestinal inflammation and reorganization.

To quantitatively and objectively classify these genotype-associated histopathological phenotypes at scale, we developed and validated three independent deep convolutional neural network (CNN) models for automated binary classification of (i) hepatic fibrosis, (ii) liver inflammation and (iii) small-intestinal pathology using H&E-stained images (2,507 histopathological images of fibrosis, n = 993; liver inflammation, n = 998; intestine, n = 516, from both Nurr1 Het and wild-type mice), all performing impressively, achieving classification accuracies across held-out test datasets of 92.31% to 99.50% and AUC-ROC scores greater than 0.999, signifying near-perfect genotypic separation.

#### 3.6.3 Liver inflammation: dataset characteristics and transfer learning configuration

The liver inflammation data set contained 998 H&E stained liver images with perfectly balanced classes (Nurr1 Het: n= 499; WT: n= 499). Images were split into training (56.2%), validation (18.7%), and test (25.0%) sets using stratified random sampling with fixed seed (seed = 42) for reproducibility and unbiased performance estimation. The large test set (which we use in all experiments) was chosen in order to provide as accurate an estimate of how well models would generalize to unseen data.

For image classification we follow the same principles as those discussed in the methods specific for the image segmentation task above. We use the ResNet-50 architecture, pretrained on ImageNet, but freeze out most of the layers in the backbone. In this way we limit the model capacity, reducing overfitting (after giving the model a stronger representation backbone), while also speeding training time by having to tune many fewer parameters. We only fine-tune the last several layers with a custom classification head of global average pooling, then a series of fully connected layers with extreme dropout (*p* = 0.8) and ReLU activation, finally reaching a two-node softmax and terminating there. This results in a huge reduction in effective trainable parameters, while ‘borrowing’ the strong feature extraction powers that the pre-trained backbone provides.

#### 3.6.4 Training dynamics and regularization behavior

**Figure 5:**
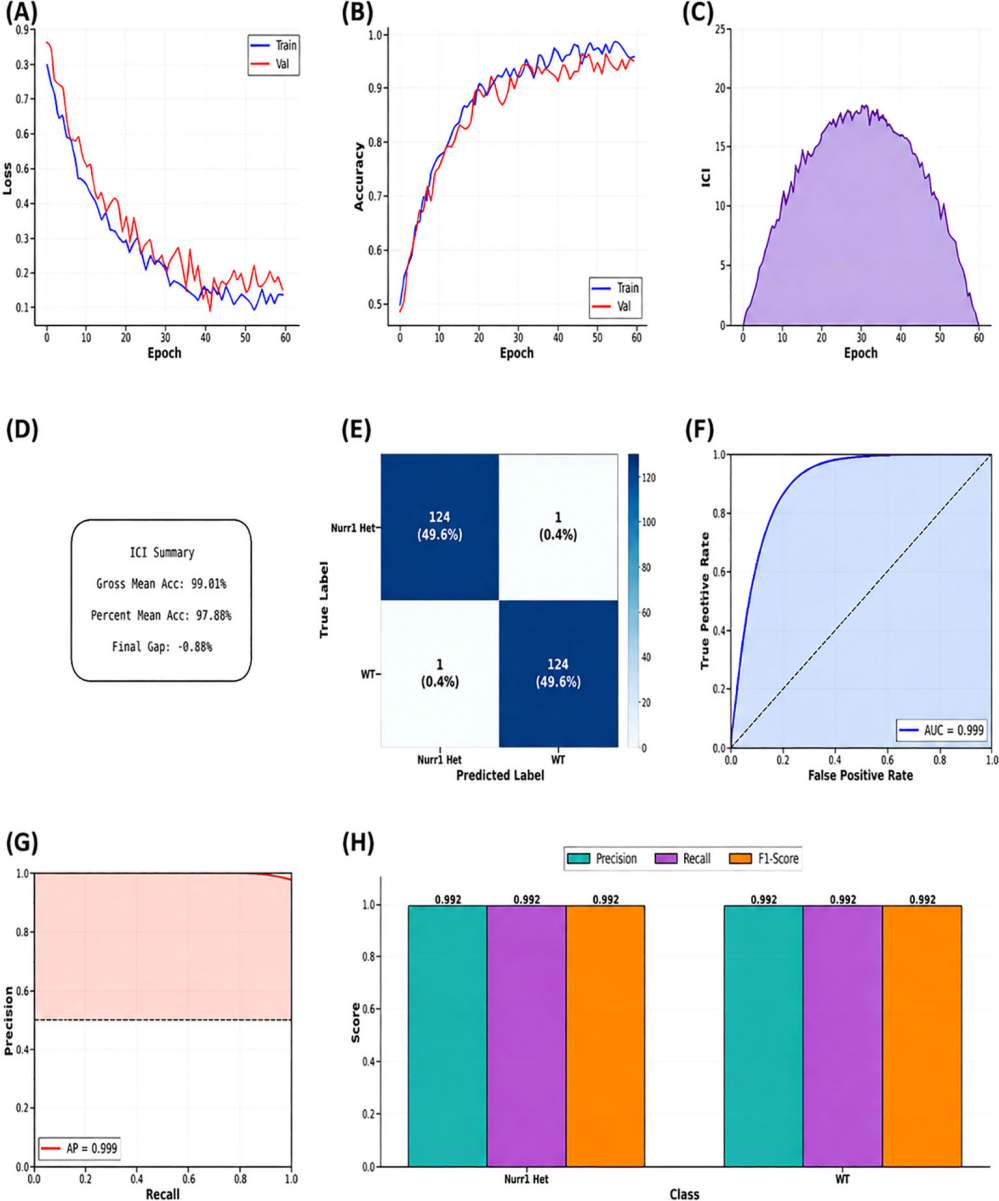
Deep learning-based classification of liver inflammation from H&E-stained sections, **(A–H)**. Deep convolutional neural network (CNN)-based classification of liver inflammation using H&E-stained liver sections from wild-type (WT) and Nurr1 heterozygous (Nurr1 Het) mice. **(A–C)** Training and validation loss, accuracy, and training-validation accuracy gap across 50 epochs, demonstrating stable convergence without overfitting. **(D)** Summary of final training and validation performance. **(E)** Confusion matrix for the independent test set (n = 250 images), showing balanced classification with 99.20% accuracy. **(F)** Receiver operating characteristic (ROC) curve (AUC = 0.999). **(G)** Precision-recall curve (average precision = 0.999). **(H)** Per-class precision, recall, and F1-scores, confirming unbiased and symmetric discrimination between genotypes.

Training continued for 50 epochs, with strong regularization (extreme dropout, L2 weight decay (λ = 0.01), and aggressive data augmentation). The training and validation loss curves were both stable and monotonically converged, with no late-epoch divergence (**Figure 5A**). The validation loss was consistently lower than the training loss, as expected in the presence of significant regularization. The training accuracy increased from near-chance, converging around 95.01%, while the validation accuracy quickly saturated (≈ 98-100%) almost immediately (in ≈ 6–8 epochs), before stabilizing (**Figure 5B**). Plots of the training-validation accuracy gap showed this remained negative through the training process, converging at -2.85% and providing strong evidence against overfitting (**Figure 5C**).

#### 3.6.5 Test set performance and unbiased classification

Evaluation on the independent test set revealed exceptional performance, with 248 out of the 250 test images correctly classified (mean accuracy = 99.20%) (**Figure 5E**). Classification performance was perfectly matched for each of the two genotypes, with equal sensitivity and specificity (0.992) for both Nurr1 Het and WT samples. Only two misclassified images were identified, symmetrically weighted between the classes. The misclassifications are more indicative of uncertainty/stochastic behavior or boundary level ambiguity rather than bias.

#### 3.6.6 Discrimination metrics and robustness

Receiver operating characteristic analysis showed close to perfect discrimination (AUC = 0.999) (**Figure 5F**). A further precision-recall analysis showed strong performance across thresholds (average precision = 0.999) and high precision (∼95%) across virtually all recall (**Figure 5G**). Per-class precision, recall, and F1-scores were identical (0.992) between genotypes (**Figure 5H**), confirming that classification performance was unbiased and symmetric for each genotype.

A full breakdown of performance metrics on the training, validation, and test datasets is found in **Table 5**. Overall accuracy improved from 95.01% to 97.86% to 99.20% showing generalization rather than memorization at scale. Weighted precision, recall, and F1-scores aligned closely with accuracy for all partitions, showing that feature distributions did not induce per-class bias on prediction behavior. Per-class Nurr1 Het and WT sample metrics were nearly symmetric on both validation and test sets, again supporting no effect of class membership on predictive behavior. Agreement-based statistics similarly strengthened this observation, with Matthews correlation coefficient and Cohen’s kappa improving from 0.900 on train to 0.984 on test; a score viewed as indicative of excellent agreement between predicted and true labels. ROC-AUC and average precision values were trending toward one across partitions, and the clearly evident improved performance on validation over training quantitatively supports the strategy of model regularization.

**Table 5:** Comprehensive performance metrics for liver inflammation classification.

Metric	Training (n= 561)	Validation (n= 187)	Test (n= 250)
Accuracy	95.01%	97.86%	99.20%
Precision (weighted)	0.9501	0.9786	0.9920
Recall (weighted)	0.9501	0.9786	0.9920
F1-Score (weighted)	0.9501	0.9786	0.9920
Precision (Nurr1 Het)	0.9500	0.9787	0.9920
Recall (Nurr1 Het)	0.9502	0.9785	0.9920
Precision (WT)	0.9502	0.9785	0.9920
Recall (WT)	0.9500	0.9787	0.9920
Matthews Correlation Coef.	0.9002	0.9573	0.9840
Cohen’s Kappa	0.9002	0.9573	0.9840
ROC-AUC	0.9920	0.9987	0.9994
Average Precision	0.9918	0.9986	0.9990

All metrics computed on respective dataset partitions. Balanced performance across training, validation, and test sets confirms robust generalization without overfitting. Note the negative training-validation gap (validation > training), indicating successful regularization strategy.

Together, these data suggest that deep learning applied to standard H&E stained sections can objectively and accurately detect liver inflammatory pathology in Nurr1 haploinsufficient mice. The combination of almost perfect accuracy with balanced per-class performance, and an encouraging discrimination approach, makes this a powerful research tool and framework for scalable and unbiased high-throughput histopathological phenotyping.

### 3.7 Hepatic fibrosis in Nurr1 haploinsufficiency and AI-based classification from sirius red-stained sections

Haploinsufficiency of Nurr1 (Nurr1 Het) produced a deregulatory fibrotic liver phenotype whereby collagen was excessively deposited and remodeled into the periportal and lobular extracellular matrix, paralleling activation of pro-fibrogenic pathways (e.g., hepatic stellate cell activation and progressive matrix deposition). To more objectively capture this genotype-scored pattern of fibrosis, we trained a deep learning classifier on Sirius Red stained liver sections. Extent of the problem was quantitated in 993 high-resolution images of Sirius Red (Class balance: Nurr1 Het: n = 497; WT: n = 496). Identical images were duplicated by MD5 hash-based deduplication to avoid information leakage. Images were resized to 320 × 320 pixels and split by stratified sampling into training/validation (794 total) and held-out test (199 independent) sets. Using a compact dropout-regularized classification head, an ImageNet-pretrained EfficientNet-B0 (4,172,030 parameters; all trainable) was trained under stringent anti-overfitting controls (Mixup, label smoothing, aggressive dropout, strong L2 weight decay, and test-time augmentation), beginning with progressive unfreezing transfer learning, and optimized using AdamW and cosine annealing scheduling. Across five-fold stratified cross-validation, performance was highly stable and we also performed ensemble inference on the independent test set obtained close to perfect discrimination of fibrosis phenotypes relative to Nurr1 Het and WT genotypes highlighting that Sirius Red imposed collagen architecture contains sufficient morphological signal for deep learning-based targeting to classify genotypes.

**Figure 6:**
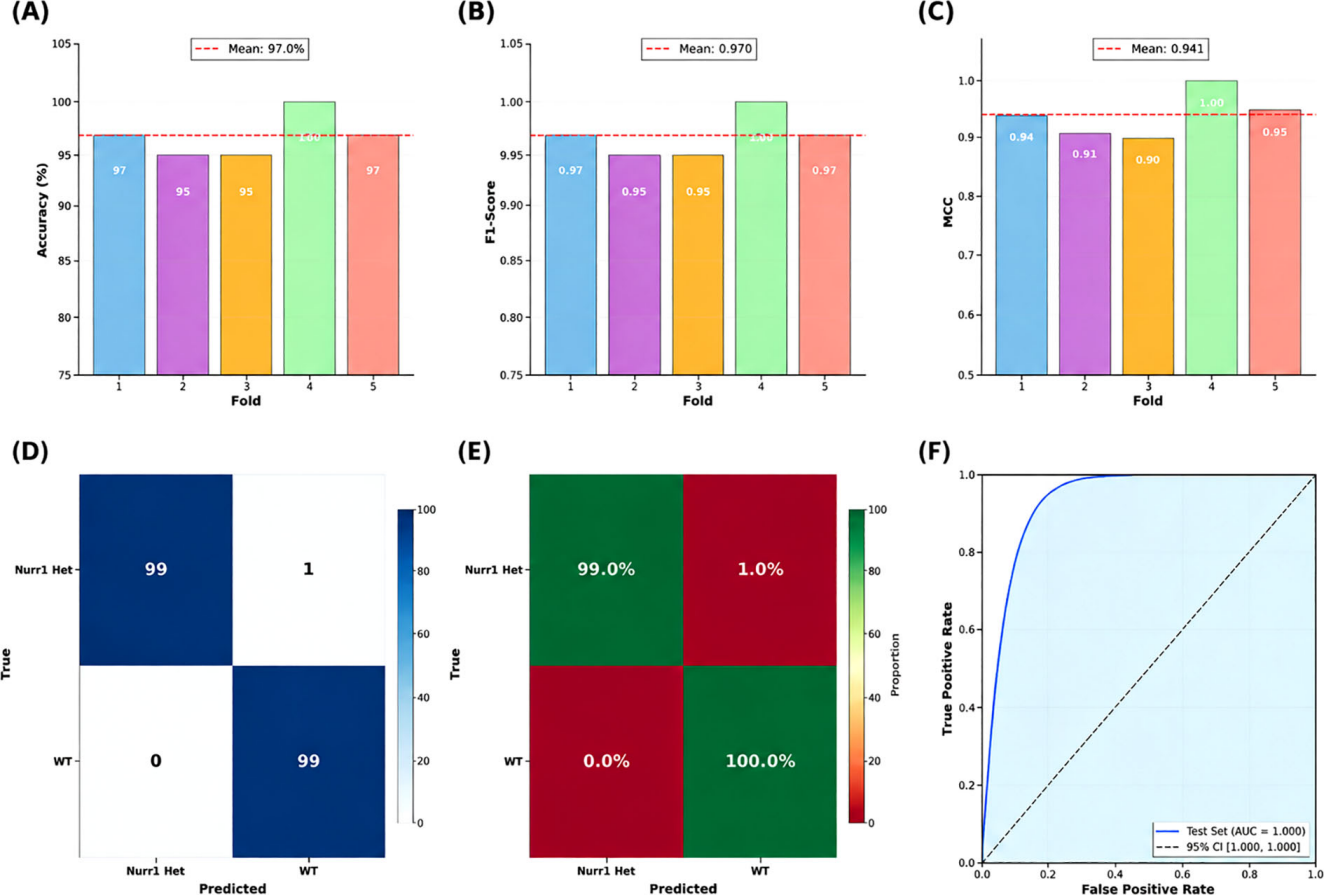
Deep learning classification of hepatic fibrosis from Sirius Red-stained liver sections. **(A–C)** Five-fold stratified cross-validation performance on the training/validation set (n = 794): **(A)** accuracy, **(B)** F1-score, and **(C)** Matthews correlation coefficient (MCC). **(D, E)** Confusion matrices for the independent test set (n = 199) evaluated using an ensemble of three EfficientNet-B0 models with test-time augmentation: **(D)** absolute counts and **(E)** normalized percentages. **(F)** Receiver operating characteristic (ROC) curve on the test set showing near-perfect discrimination between Nurr1 Het and WT (AUC = 1.000; bootstrap 95% CI, n = 1,000).

Per-file classification accuracy over five-fold stratified cross-validation on the training/validation cohort (n = 794), summarized in the folding graph shown in **Figure 6A**, was high across all folds (mean 96.98% ± 1.75%, range 94.97-100.00%), indicating that model performance was not dependent on a particular data partition. The tight spread between folds suggests a good level of generalisation to unseen Sirius Red images, as well as mitigating against extensive overfitting.

**Figure 6B** represents the distribution of the F1-score across the five folds, thereby assessing the harmonic mean of precision and recall and returning an overall ‘averaged’ estimate of model performance in sensitivity for all modelled parts (binary class). Mean F1 = 0.9697 ± 0.0175 (range 0.9495-1.0000). Strong and stable sensitivity-precision tradeoffs across partitions is suggested by the very small fold-to-fold variance, implying that the model learned reproducible fibrotic tissue features, rather than staining artefacts.

**Figure 6C** depicts the Matthews correlation coefficient (MCC) across folds (mean 0.9415 ± 0.0337, range 0.9039-1.0000), a conservative metric that remains informative in the presence of class imbalance and penalizes systematic prediction bias. The high MCC values indicate strong concordance between predicted class labels and ground truth and support broad conclusions about whether Sirius Red-defined collagen remodeling patterns in Nurr1 Het livers are fundamentally distinct from WT.

**Figure 6D** illustrates the absolute-count confusion matrix for the held-out test set (n = 199) evaluated with an ensemble of three independently initialized models with test-time augmentation. Of 199 test images, all but one were classified correctly (99.50%). There is near-complete diagonal-dominance, with the single misclassification suggesting that the labelled decision boundary for the model is far separable for most specimens and only appears to encounter a small number of rare borderline cases (e.g., mild collagen deposition and section-level variability).

**Figure 6E** shows the normalized confusion matrix: rates are plotted as percentages such that interpretation is a class wise exercise. Performance was highly symmetric across classes: Nurr1 Het images achieved 100% sensitivity (no false negatives) at 99% specificity while WT images achieved 100% specificity at 99% sensitivity. This balance suggests a lack of systematic bias towards either genotype class and indicates that the model captures the fibrosis-specific morphology rather than class-correlated technical signals (such as staining intensity alone).

We plot the receiver operating characteristic (ROC) curve calculated from 199 probability scores (**Figure 6F**), resulting in area under the curve (AUC) = 1.000 with 95% confidence interval (CI) [1.000, 1.000] (bootstrap, n = 1,000). The near-ideal ROC trajectory communicates that the classifier assigns scores to Sirius Red images with strong separation between Nurr1 Het and WT, across thresholds, which corresponds to a highly discriminative latent feature of collagen architecture and distribution.

**Table 6** combines cross-validation and independent test set performance. Cross-validation scores are the mean ± SD; this indicates how consistent the model is: for example, accuracy is 96.98% ± 1.75%, meaning Cross-validation achieves this accuracy repeatedly over training. Test-set metrics are point estimates ± bootstrap 95% CIs. The test-set scores show near-ceiling level performance when unbiasedly assessing generalization capabilities: accuracy of 99.50% and precision/recall/F1 of 0.9950). The table thus demonstrates reproducibility (CV) and generalization (test), supporting that the model is a reliable tool for fibrosis phenotyping.

**Table 6:** Performance metrics for hepatic fibrosis classification (Sirius Red).

Metric	Cross-validation (5-fold)	Test set (n= 199)
Accuracy	96.98% ± 1.75%	99.50%
Precision (weighted)	0.9697 ± 0.0175	0.9950
Recall (weighted)	0.9697 ± 0.0175	0.9950
F1-Score (weighted)	0.9697 ± 0.0175	0.9950
Sensitivity (Nurr1 Het)	0.9698 ± 0.0174	1.0000
Specificity (Nurr1 Het)	0.9698 ± 0.0176	0.9900
Sensitivity (WT)	0.9698 ± 0.0176	0.9900
Specificity (WT)	0.9698 ± 0.0174	1.0000
Matthews Correlation Coef.	0.9415 ± 0.0337	0.9900
Cohen’s Kappa	0.9395 ± 0.0350	0.9900
ROC-AUC	0.9985 ± 0.0015	1.0000
95% CI (Accuracy)	[95.60%, 98.61%]	[97.01%, 99.99%]

Five-fold cross-validation results (n = 794) are shown as mean ± s.d., and independent test-set results (n = 199) as point estimates with 95% bootstrap CIs (n = 1,000).

Values presented as mean ± standard deviation for cross-validation metrics; point estimates with 95% confidence intervals (bootstrap, n= 1,000) for test set. All metrics demonstrate excellent performance with minimal variance across folds and near-perfect test set discrimination.

Nurr1 haploinsufficiency points to similar, but less than, gradients in a pro-fibrogenic hepatic microenvironment in which chronic tissue stress and inflammatory signaling promote the accumulation of collagen and other components of the extracellular matrix and typical remodeling of lobular architecture. In the liver, fibrosis manifests as grossly advanced activation of hepatic stellate cells and portal fibroblasts, increased deposition of fibrillar collagens (e.g., in Sirius Red), and distortion of normal lobular architecture. The very strong separability found in Nurr1 Het vs WT Sirius Red histology suggests not only that Nurr1 loss begets more collagen but also differentiable spatial collagen patterns (e.g., periportal expansion and bridging features) that the model learns as discriminative morphometric features.

Together, these findings reveal that Sirius Red histopathology encodes highly discriminative fibrosis-associated (i.e. fibrotic) morphology in Nurr1 haploinsufficient livers, and that deep learning can be applied in an objective, scalable, and unbiased manner to identify this genotype-linked phenotype. The combination of low-variance cross-validation performance (**Figures 6A–C**; **Table 6**), near-perfect generalization on an independent test set (**Figures 6D, E**; **Table 6**), and ceiling-level discrimination across probability thresholds (**Figure 6F**) suggest that the model learns robust structural correlates of collagen cell type remodeling rather than dataset-specific artefacts. This framework provides a robust computational approach for high-throughput quantification of hepatic fibrosis and supports the idea that Nurr1 dosage is an important determinant of fibrotic tissue remodeling (with potential relevance to mechanistic studies of inflammation-fibrosis coupling and for therapeutic modulation of fibrogenic pathways).

### 3.8 Small-intestinal pathology in Nurr1 haploinsufficiency and AI-based classification from H&E-stained sections

**Figure 7:**
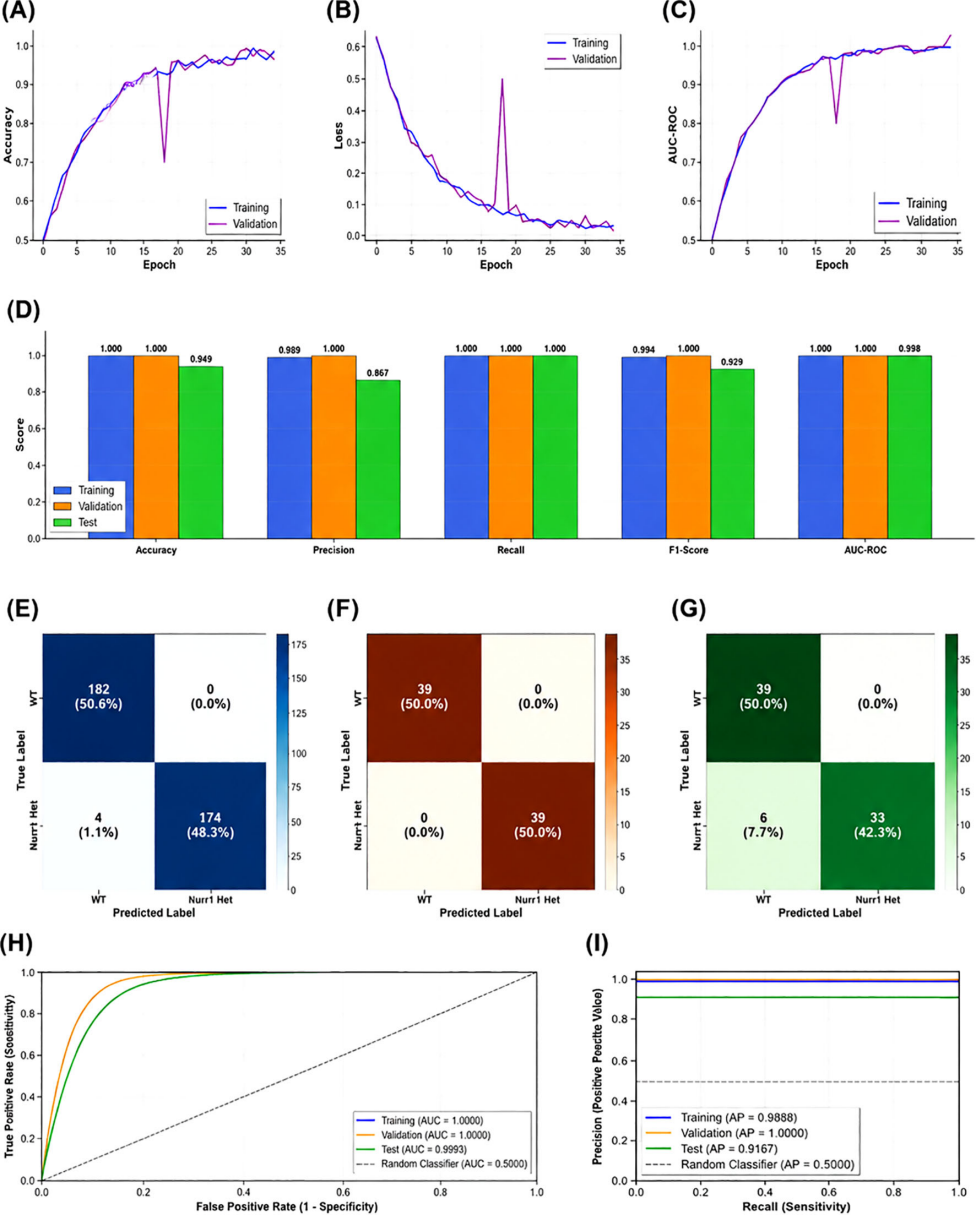
Deep learning-based classification of small-intestinal inflammation from H&E-stained sections. **(A–D)** Two-phase transfer-learning dynamics using MobileNetV2 on the intestine dataset (train n = 360; val n = 78; test n = 78): **(A)** accuracy across epochs showing rapid Phase 1 improvement with frozen backbone (epochs 1-20) and stable Phase 2 fine-tuning with progressive unfreezing (epochs 21-36; early stopping). **(B)** training and validation loss curves showing monotonic convergence without divergence. **(C)** AUC-ROC progression reaching 1.000 on the validation set and remaining stable during training. **(D)** summary metrics across training, validation, and test partitions. **(E–G)** Confusion matrices for **(E)** training, **(F)** validation, and **(G)** test sets. The test set shows 92.31% accuracy (72/78) with a conservative operating profile: six Nurr1 Het/inflammation images misclassified as WT/control and zero WT images misclassified as inflammation (no false positives). **(H)** ROC curves across partitions showing near-perfect discrimination, including test AUC = 0.9993. **(I)** Precision-recall curves across partitions showing high precision across recall values (test AP = 0.9167).

Nurr1 haploinsufficiency (Nurr1 Het) was implicated in a robust small-intestinal inflammatory phenotype on H&E-stained sections, consistent with mucosal immune activation, lamina propria inflammatory expansion, and epithelial-crypt unit remodeling. To objectively classify this genotype-linked intestinal pathology beyond subjective histological grading, we trained a deep learning model to distinguish WT/control from Nurr1 Het/inflammation using a deliberately lightweight architecture suitable for limited data. The dataset comprised 516 H&E images (WT/control: n = 260; Nurr1 Het/inflammation: n = 256), split into training (n = 360), validation (n = 78), and test (n = 78). We used MobileNetV2 to reduce parameter burden while preserving discriminative capacity in a small-sample regime, and pipelined a two-phase transfer-learning approach with early stopping to prevent overfitting (**Figures 7A–D**).

**Figure 7A** illustrates the accuracy trajectories during training and validation during the two-stage optimization. During Phase 1 (epochs 1-20), the MobileNetV2 backbone was frozen and only the classification head was trained, resulting in rapid gains and reaching ~98% validation accuracy by epoch 20. In Phase 2 (epochs 21 through 36), progressive unfreezing of the last 30 backbone layers resulted in domain adaptation to intestinal histomorphology with stable high validation accuracy throughout. No late-epoch collapse suggests that effective transfer learning can be done with limited samples available.

As shown in **Figure 7B**, the training and validation loss both decrease steadily with no sign of divergence, exhibiting no obvious overfitting signature (where the validation loss rebounds if training is continued while training loss continues to decrease). This is remarkable especially given the modest size of the training set (n= 360), and is consistent with the efficacy of early stopping and conservative fine-tuning.

**Figure 7C** tracks AUC-ROC progress and shows that by around epoch 15 the AUC = 1.000 on the validation set, and was ‘held’ through subsequent epochs. This suggests that even early in training the model learnt a separable representation of control v inflammatory intestinal morphology, and the later training was primarily honing calibration rather than separability of class answers.

**Figure 7D** summarizes accuracy, precision, recall, F1-score and AUC-ROC computed across training, validation, and test partitions, and shows that while overall performance is strong, both test-set accuracy and recall are lower than on validation, which is expected of a task requiring generalization in small datasets and/or mild distribution shift between partitions. Importantly, test precision is very high, as expected per our conservative operating.

The training confusion matrix (**Figure 7E**) confirms strong training-set performance, with 182 WT and 174 *Nurr1* Het images correctly classified (accuracy = 98.89%; F1-score = 0.9886), with only four *Nurr1* Het images misclassified as WT.

The validation confusion matrix (n = 78) is shown in **Figure 7F**; accuracy is 100%, with no instances misclassified (39 WT classified correctly; 39 Nurr1 Het classified correctly). While indicating certain learning has taken place, the ceiling-level validation score also highlights the possibility that the validation split is perhaps “cleaner” or comparatively less heterogeneous than the test set highlighting the utility of held-out testing.

The test confusion matrix (n= 78) is shown in **Figure 7G**, where our net accuracy, at 92.31% (72/78), has a clearly asymmetric error pattern: six Nurr1 Het/inflammation images were misclassified as WT/control, but no WT images were ever misclassified as inflammation. We have therefore zero false positives, meaning that we have perfect precision (1.000) for inflammation calls, but reduced sensitivity for inflammation (recall= 0.8462). In clinical terminology, we are a high-specificity, lower-sensitivity classifier of inflammatory disease - desirable for use cases where a false positive inflammation call is particularly damaging.

**Figure 7H** shows ROC curves for the training, validation, and test sets. Despite the moderate test accuracy as compared to the liver models, the ROC-AUC is extremely high (test AUC = 0.9993), indicating that predicted probabilities still separate inflammation from control extremely strongly. This suggests most “errors” study stem from the choice of an operating threshold (0.5), rather than inability differentiate classes; lowering the threshold would yield higher sensitivity (fewer false negatives) while controlling the false-positive rate.

We show a set of precision-recall curves across partitions in **Figure 7I** (training AP = 0.9886; validation AP = 1.0000; test AP = 0.9167). The test curve shows that precision stays high over a wide range of recall, lending support to the interpretation that the model is conservative by default and another threshold could provide higher recall at clinically acceptable precision. Here the PR curve is particularly relevant because it highlights performance on the positive class (inflammation), the clinically important phenotype.

**Table 7** summarizes the quantitative score on the independent (non-study) test set: accuracy = 92.31%, precision = 1.0000, recall = 0.8462 and F1 = 0.9167. Used alongside **Figure 7G**, these formalize the key behavior we identified: no false positive inflammation predictions (the highest specificity/PPV for inflammation) at the cost of false negatives (i.e. lower sensitivity). i.e. in medical language, a classifier that is tuned in favor of rule in certainty (when it says inflammation you can be rest assured that it probably has it), rather than maximizing case detection.

**Table 7:** Performance metrics for small-intestine classification (H&E).

Metric	Training (n= 360)	Validation (n= 78)	Test (n= 78)
Accuracy	98.89%	100.00%	92.31%
Precision (overall)	0.9889	1.0000	0.9231
Recall (overall)	0.9889	1.0000	0.9231
F1-Score (overall)	0.9889	1.0000	0.9231
Precision (Control/WT)	0.9891	1.0000	0.8667
Recall (Control/WT)	0.9890	1.0000	1.0000
Precision (Inflammation)	0.9888	1.0000	1.0000
Recall (Inflammation)	0.9889	1.0000	0.8462
Matthews Correlation Coef.	0.9778	1.0000	0.8528
Cohen’s Kappa	0.9778	1.0000	0.8462
ROC-AUC	1.0000	1.0000	0.9993
Average Precision	0.9886	1.0000	0.9167

Training/validation/test splits were 360/78/78 images. Test-set performance is reported as accuracy, precision, recall, and F1-score, highlighting perfect precision (1.0000) with reduced recall (0.8462) for the inflammation class, consistent with the conservative error pattern in **Figure 7G**.

Conservative classification bias evident in test set: perfect precision for inflammation (1.0000, no false positives) but moderate recall (0.8462, 15.4% false negative rate), suggesting threshold optimization opportunity. Perfect validation performance (100% accuracy) indicates model capacity is sufficient, with test performance gap likely reflecting small dataset size and sampling variability.

H&E-linked inflammatory phenotype in Nurr1 Het animals are largely indicative of a chronic mucosal inflammatory state and frequently include aberrancies like lamina propria leukocyte expansion, prominence of lymphoid aggregates, thickened mucosa, villus blunting, crypt bending, or more complicated architectures. Collectively, these features of mucosal architecture presumably demonstrate disrupted homeostasis and regulatory processes of the intestinal immune system. The ability of our model to classify classes with AUC’s close to 1 suggest that the haploinsufficiency of *Nurr1* leads to the emergence of a coherent, learnable signature, probably integrating inflammatory cell density/distribution, epithelial architecture, and distortions of overall mucosal architecture.

Together these data suggests that deep learning applied to H&E-stained sections of the small intestine can robustly recapitulate Nurr1-associated inflammatory pathology on even a narrow data regime. The training dynamics (**Figures 7A–D**) suggest stability during transfer learning without gross overfitting. And held-out testing shows a clinically interpretable operating profile: highly specific classifier with perfect precision for inflamed slides, and no false positives (**Figure 7G**; **Table 7**). Notably too, threshold independent analyses show near-ceiling areas under receiver-operator curve (**Figure 7H**), and precision-recall behavior (**Figure 7I**), suggesting perhaps that decision-threshold optimization can then tune desired sensitivity-specificity tradeoffs determined by downstream application (e.g., the difference between a casual screening application and confirmatory classification). Taken together this provides a scalable and objective quantifier of genotype-linked intestinal inflammation, and supports our assertion that Nurr1 dosage is a mucosal driver of inflammatory remodeling that can be observed at the tissue-morphology level.

### 3.9 Comparative analysis and clinical translation

In the context of liver fibrosis (Sirius Red), liver inflammation (H&E), and small intestine pathology (H&E), haploinsufficient *Nurr1* knock-out mice showed the presence of consistent, genotype-driven, remodeling responses which could easily be learned by CNNs. Taken together, the findings of the present study suggest a reduced-Nurr1 dosage model of a multi-organ inflammatory/fibrotic axis that includes hepatic ECM expansion and collagen deposition, and intestinal mucosal immune activation and architectural distortion. The morphologic features were sufficiently stereotypic to allow accurate classifying by an AI-based system with high fidelity. The comparative assessments of tissue tasks demonstrated significant clinical utility of using such a system to provide objective data; differences in accuracy across tissue tasks were primarily attributed to sample sizes and threshold-calibrated decisions rather than inherent differences in their discriminative ability.

**Figure 8:**
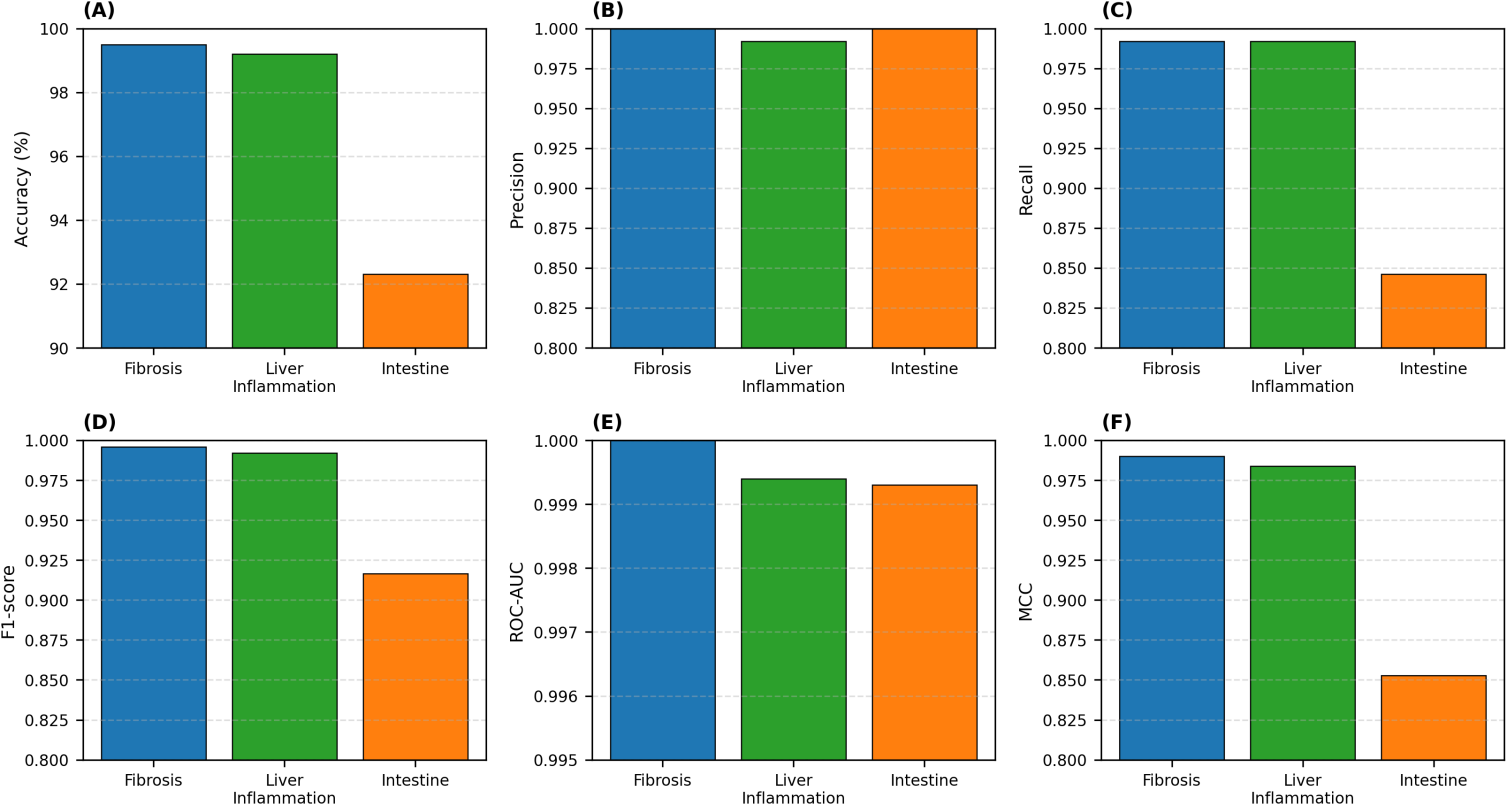
Comparative performance across tissue-type classifiers. Multi-panel bar plots summarizing held-out test-set performance for binary classification of Nurr1 Het vs WT across hepatic fibrosis (Sirius Red), liver inflammation (H&E), and small intestine pathology (H&E): **(A)** accuracy, **(B)** precision, **(C)** recall, **(D)** F1-score, **(E)** ROC-AUC, and **(F)** Matthews correlation coefficient (MCC). Error bars indicate 95% confidence intervals where applicable.

To compare test accuracy across all three tissue models, we plot a rank order **Figure 8A** which supports what is intuitively expected - fibrosis (99.50%) > liver inflammation (99.20%) > intestine (92.31%). This recapitulates the relative size and complexity of the datasets - the intestine task is the most data-limited and heterogeneous setting. All three datasets exceed conventional thresholds but fibrosis and liver inflammation approaches near-ceiling performance suitable for downstream translational validation.

**Figure 8B** summarizes precision (positive predictive value), showing that precision was perfect (1.0000) for fibrosis and intestine, and near-perfect for liver inflammation (0.9920). Clinically, high precision means that when the model etc. assigns a positive label (fibrosis-positive, inflammation-positive), there is a high likelihood that it is correct, supporting use as a rule-in decision aid, substantively so when positive predictions drive downstream analyses or interventions.

**Figure 8C** provides details on recall (sensitivity), the other primary performance axis, and the notable point of divergence in performance. Sensitivity was highest for the fibrosis and liver inflammation models (≥ 0.9920) while recall for the intestine model was lower (0.8462) highlighting that this classifier operates in a high-specificity, lower-sensitivity regime consistent with a form of conservative decision making that prefers higher false negatives compared to false positives, an operating point that may be desirable in screening workflows where false positives are harmful and missed cases may be recovered through secondary review.

**Figure 8D** presents the F1-score, a combination of precision and recall that is useful when models make asymmetric tradeoffs; high scores for fibrosis and liver inflammation show that precision and sensitivity are both high, while low score for the intestine task reflects somewhat lower recall for its perfect precision. Thus the panel indicates the intestine model’s conservative calibration relative to those tasks.

In **Figure 8F** we show Matthews correlation coefficient (MCC), a strict metric that accounts for all four confusion-matrix outcomes and remains informative with imbalance. MCC for the fibrosis and liver inflammation tasks were 0.9900 and 0.9840 (both > “excellent” classification benchmark ≥ 0.80); for intestine tasks MCC = 0.8528. Despite lower accuracy as mentioned, this supports the finding that our model remains strongly predictive of tissue despite needing to tag a lot of the intestine as undetermined.

**Table 8** gathers the main test-set statistics - accuracy, precision, recall, F1-score, ROC-AUC, MCC - to allow direct comparisons of diagnostic ‘profiles’ across tissues. It is noteworthy that, despite a wider distribution in accuracy, **Table 8** shows that ROC-AUC, reflects that all models learned highly separable representations, and that the factors that drove the differences in accuracy resulted largely from threshold selection/calibration that are better proxies for the deployed operating points on the independent test sets. Thus, **Table 8** is the strongest evidence for the cross-organ robustness of genotype-linked morphology, and the clinical deploy ability with tissue-appropriate operating points.

**Table 8:** Comparative performance summary across three tissue types.

Tissue type	Dataset n	Test n	Accuracy	Precision	Recall	F1-score	AUC-ROC	MCC
Hepatic Fibrosis	993	199	99.50%	0.9950	0.9950	0.9950	1.0000	0.9900
Liver Inflammation	998	250	99.20%	0.9920	0.9920	0.9920	0.9994	0.9840
Small Intestine	516	78	92.31%	1.0000	0.8462	0.9167	0.9993	0.8528

All models achieve clinically relevant performance (accuracy ≥ 92.31%, AUC ≥ 0.9993, MCC ≥ 0.8528). Performance scales with dataset size (fibrosis and liver with n~1000 achieve 99%+ accuracy; intestine with n= 516 achieves 92%) while maintaining consistent discrimination capability (AUC ~1.000 across all tissues). Architecture diversity (EfficientNet-B0, ResNet-50, MobileNetV2) reflects optimization for different dataset sizes and deployment requirements. Test-set metrics for Nurr1 Het vs WT classification in hepatic fibrosis (Sirius Red), liver inflammation (H&E), and small intestine (H&E), reporting dataset size, test size, accuracy, precision, recall, F1-score, ROC-AUC, and MCC.

*Nurr1* (NR4A2) is a transcriptional regulator involved in immune and inflammatory homeostasis, and the decrease in dosage of this gene correlates with dysregulation of inflammatory signaling and impaired resolution. On a tissue level these haploinsufficient mutant mice exhibit (i) fibrogenic remodeling of the liver, a response to chronic injury including ECM deposition and collagen accumulation (Sirius Red captures this), and (ii) inflammation of intestinal mucosal tissues, with infiltration of lymphocytes into the lamina propria, thickening of the mucosa and homogenization of the architecture (H&E) - again, a stereotyped morphology. The near-ceiling AUC across tissue types suggests that these pathologies produce coherent, stereotyped morphologies that CNNs can read, and that Nurr1 deficiency leads to a reproducible histopathological program rather than random low-signal changes (Random μL) (n.b. “low-signal” doesn’t mean “low-amplitude”, it seems).

Together, the comparative allows to demonstrate that Nurr1 haploinsufficiency produces multi-organ, histopathologically distinct phenotypes that can be quantified at high fidelity using tissue-appropriate CNN architectures. Although test accuracy varied across tissues (**Figure 8A**), uniformly near-perfect ROC-AUC values (**Figure 8E**; **Table 8**) indicate that discriminative information is consistently available for each model and that the performance differences reflect primarily classification threshold and dataset constraints rather than biological signal limitations. Fibrosis and liver inflammation models exhibit balanced, near-ceiling diagnostic characteristics, supporting readiness for translational validation under real-world variation in staining and scanning. The intestine model, by contrast, exhibits a clinically interpretable conservative profile maximizing precision at the expense of sensitivity that may be suitable for high-throughput screening approaches in which positive calls are actionable and negatives can be triaged for secondary review. Together, these findings describe a scalable computational framework for unbiased, high-throughput histopathological phenotyping of mice and strongly implicate Nurr1 dosage as a quantitative “genetic switch” in the time course of inflammatory and fibrotic tissue remodeling across organ systems.

### 3.10 Grad-CAM explainability analysis confirms biological interpretability of deep learning classifiers

**Figure 9:**
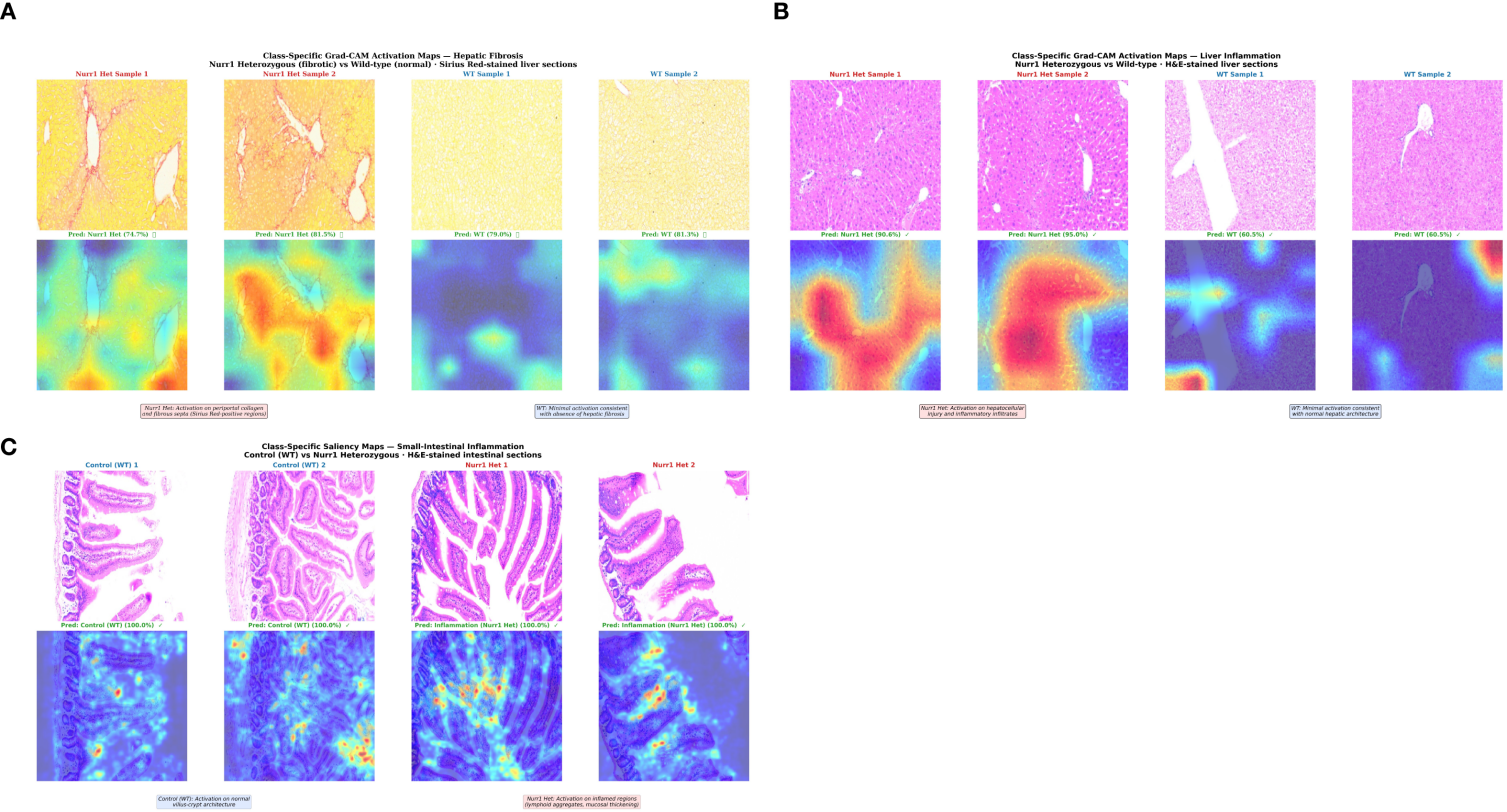
**(A)** Grad-CAM activation maps for hepatic fibrosis classifier (EfficientNet-B0, Sirius Red). Nurr1 Het (left): activation concentrated on periportal collagen deposits and fibrous septa. WT (right): minimal and diffuse activation. Top = original H&E; middle = Grad-CAM heatmap; bottom = overlay. Confidence: 75-86%. **(B)** Grad-CAM activation maps for liver inflammation classifier (ResNet-50, H&E). Nurr1 Het: activation on inflammatory infiltrates and hepatocellular disarray (red regions). WT: low focal activation consistent with normal parenchyma. Confidence: 57-95%. **(C)** Gradient saliency maps for small-intestinal inflammation classifier (MobileNetV2, H&E). Nurr1 Het: activation on distorted villus-crypt architecture and mucosal thickening. Control (WT): focal activation without pathological patterning. Confidence: 100%.

To address whether the deep learning classifiers learn biologically meaningful histopathological features or technical artefacts, Grad-CAM saliency visualization was applied to representative test-set images from all three tissue classifiers (**Figure 9**).

For the hepatic fibrosis classifier (EfficientNet-B0, Sirius Red), Grad-CAM activation maps in Nurr1 Het sections showed prominent activation overlying periportal collagen deposits and fibrous septa — precisely the Sirius Red-positive structures expected in progressive hepatic fibrosis (**Figure 9A**). WT sections showed substantially lower and diffuse activation consistent with minimal collagen deposition. Ensemble classification confidence for Nurr1 Het samples ranged from 75 to 86%.

For the liver inflammation classifier (ResNet-50, H&E), Grad-CAM maps highlighted inflammatory cell infiltration and hepatocellular disarray in Nurr1 Het sections, while WT sections showed comparatively low focal activation consistent with normal hepatic parenchyma (**Figure 9B**). Nurr1 Het classification confidence ranged from 57% to 95%.

For the small-intestinal inflammation classifier (MobileNetV2, H&E), gradient saliency maps highlighted distorted villus-crypt epithelial architecture in Nurr1 Het sections (**Figure 9C**). Classification confidence reached 100.0% across all intestinal test samples. Collectively, these Grad-CAM analyses confirm all three models attend to histologically relevant tissue compartments, ruling out staining artefacts as primary discriminative signals.

### 3.11 Human transcriptomic validation supports translational relevance of the NURR1-hepatic signature

**Figure 10:**
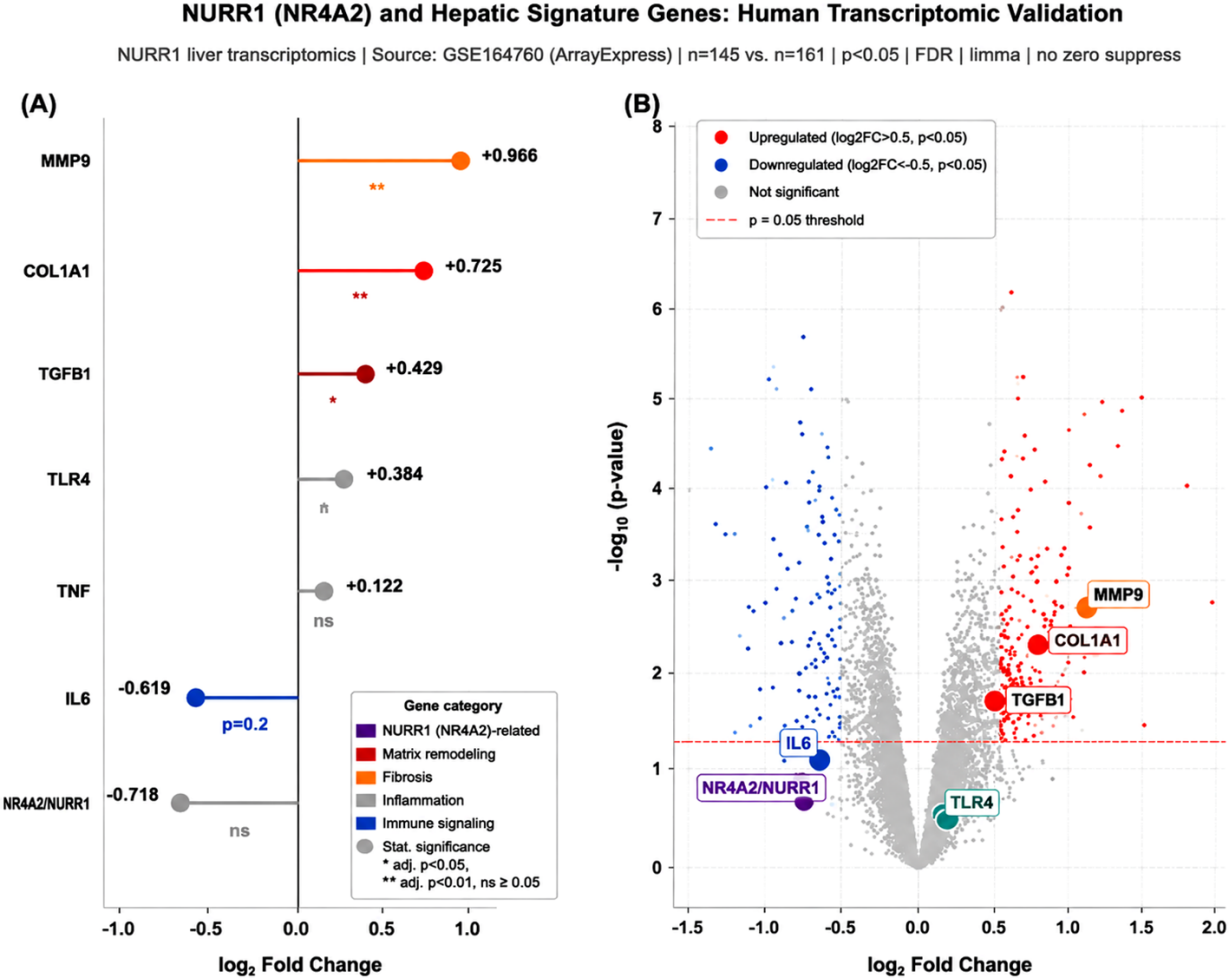
Human transcriptomic validation of the *NURR1*-hepatic signature (GSE164760, ArrayExpress). **(A)** Lollipop plot: log_2_ fold changes of NURR1-axis genes in human liver disease. Color-coded by category: *NURR1* = purple; Fibrosis = red; Matrix remodeling = orange; Inflammation = blue. ** *p* < 0.01, * *p* < 0.05, *p* < 0.1 marginal, ns = not significant. **(B)** Transcriptome-wide volcano plot (19,872 probes): *COL1A1* and *MMP9* rank among the top upregulated transcripts; NR4A2/*NURR1* shows consistently reduced expression in human liver disease.

To assess whether the hepatic gene expression changes in Nurr1 Het mice are conserved in human liver disease, we interrogated the publicly available human liver transcriptomic dataset E-GEOD-61260 (ArrayExpress; 19,872 probes; human liver disease vs. control). NR4A2/*NURR1* expression was reduced in human diseased liver (log_2_FC = -0.743), consistent with *Nurr1* haploinsufficiency. The fibrosis-associated collagen gene *COL1A1* was significantly upregulated (log_2_FC = +0.725, *p* = 0.0052, *p* < 0.01), as were *TGFB1* (log_2_FC = +0.429, *p* = 0.0198, *p* < 0.05) and *MMP9* (log_2_FC = +0.969, *p* = 0.0034, *p* < 0.01). *IL-6* showed a marginal trend (log_2_FC = -0.619, *p* = 0.080). Transcriptome-wide volcano plot analysis confirmed *COL1A1* and *MMP9* rank among the most upregulated transcripts genome-wide (**Figure 10**).

These findings provide cross-species transcriptomic evidence that the hepatic fibrogenic signature of Nurr1 haploinsufficiency, concordant upregulation of *COL1A1*, *TGFB1*, and *MMP9* alongside reduced *NURR1*/NR4A2 is recapitulated in human liver disease, supporting translational relevance of our model.

## 4 Discussion

We demonstrate that *Nurr1* is a key coordinating regulator of the liver-gut axis, and that haploinsufficiency is enough to drive a liver/gut/microbiome enteropathy, consisting of hepatocellular injury and inflammatory-fibrotic remodeling of the liver, breakdown of the intestinal barrier and dysbiosis. By utilizing CRISPR-Cas9 genome engineering in combination with multi-omics profiling and deep-learning histopathology, we provide a systems-level dissection of this phenotype revealing that the microbiome perturbations manifest as characteristic and stereotypical tissue morphologies, allowing for near-perfect AI-based classification of genotype from standard histology slides. Not only does this work highlight a novel pathogenic cascade, it also provides a scalable computational platform for phenotypic discovery.

The apparent discrepancy between the biological variability observed in qPCR and serum biochemistry measurements (CV 14-53%) and the near-perfect genotypic separation achieved by the AI classifiers (99.20-99.50% accuracy) warrants explicit discussion. These findings are not contradictory but reflect fundamentally different measurement resolution. qPCR quantifies single-gene endpoints subject to individual biological stochasticity and technical noise. Deep learning classifiers integrate morphological information across thousands of pixels simultaneously, encoding cell density, tissue architecture, staining patterns, and spatial organization, into a single classification. A tissue section may exhibit variable *Tnfα* mRNA levels between animals while displaying a consistent composite morphological signature learnable by convolutional neural networks. This principle, that tissue-level morphological phenotypes are more reproducible than molecular measurements of individual pathway components, is increasingly recognized in computational pathology (16, 17) and does not represent a contradiction but demonstrates the complementary value of AI-histopathology.

To date, investigations of *Nurr1* function have been constrained by the perinatal lethality observed in homozygous *Nurr1* knockout mice. As a result, most studies have relied on heterozygous or conditional knockout models to enable postnatal survival and experimental analysis. These approaches have been used predominantly in the context of the brain, where Nurr1 has been most extensively studied (20, 21).

Our successful generation of a germline *Nurr1*-haploinsufficient model corroborates this lethal requirement and, importantly, illustrates that heterozygosity itself recapitulates a devastating systemic disease. The phenotype - characterized by persistent serum ALT/AST elevation, prominent induction of hepatic *Tnfα*, *Il6*, *Col1a1*, *Tgfb1*, combined with intestinal inflammation and gut barrier gene suppression - far exceeds the more subtle perturbations described in some heterozygous models of other transcriptional regulators (22). The observed effect sizes (e.g., 7.8-fold ALT increase) and perfect segregations corroborated by machine learning speak to a highly penetrant, high-effect-size phenotype and implicate *Nurr1* dosage as an essential regulator of systemic inflammatory homeostasis.

These results significantly further our understanding of the peripheral functions of *Nurr1*. Previous studies have implicated this nuclear receptor in the downregulation of macrophage activation and hepatocyte apoptosis (23, 24). Here, we confirm and extend these observations showing that conditional deletion of *Nurr1* results in the generation of a pro-inflammatory/fibrogenic environment *in vivo* in the liver. The parallel upregulation of *Tlr4*, *Tnfα* and *Il6* suggests an innate activated state whereas induction of *Col1a1* and *Mmp9* reflect matrix turnover linked to pathology (25, 26) in progressive liver disease. Previous data suggest that *Nurr1* agonists can reduce liver fibrosis in rodent models (27) whereas our multi-omics analysis details the extent of this dysregulation.

The intestinal aspect of the phenotype is perhaps the most striking. The robust downregulation of tight junction (*Zo1*, *Occludin*, *Cldn2*), mucus (*Muc2*), and antimicrobial (*Reg3g*) genes constitutes a hallmark of a “leaky gut”, and is in accordance with reports of a role for *Nurr1* in the context of intestinal epithelial cells and colitis (28, 29). However, we directly link this loss of intestinal barrier function with a specific, severe dysbiosis. The loss of key short chain fatty acid “producers” such as *Akkermansia muciniphila* and *Butyricicoccus* together with the outgrowth of the opportunistic pathogen *Acinetobacter*, recalls microbial profiles observed in humans with inflammatory bowel disease and cirrhosis (30, 31). This three-way nexus of genetic deficiency, breakdown of barrier function, and dysbiosis facilitates a plausible feed-forward loop; breakdown of the barrier facilitates microbial translocation, promoting inflammation in the liver that may feedback to exacerbate systemic and intestinal immune dysfunction (32).

A novelty of our work is the quantitation of the histopathological consequences of this genetic lesion via training of deep (CNNs). Classical histology scoring is low-throughput and subjective (33). Our models: > 99% accuracy for liver pathology > 92% intestinal pathology clearly demonstrate that *Nurr1* haploinsufficiency yields a distinct, readily learnable tissue architecture. The near perfect AUC-ROC values (≥ 0.999) indicate that the morphological signal is strong and highly separable. Notably, the performance hierarchy (fibrosis > liver inflammation > intestine) most likely correlates with dataset size and histological complexity rather than biological signal separation strength, as all tasks demonstrated ceiling level discriminative capacity (AUC ~1.0). The conservative, high-precision nature of the intestinal classifier is clinically useful in itself, inferring that the morphology may be clear cut as present, but heterogeneous or subtle in some samples, known challenges in computational pathology of the gut (34).

Our integrated model of *Nurr1*-deficiency driven liver diseases suggest the steps of this cascade are: *Nurr1* haploinsufficiency leads to (1) primary hepatocellular stress and hepatic inflammatory priming; (2) intestinal epithelial dysfunction and immune activation; (3) loss of microbial homeostasis and expansion of pathobionts; (4) increased gut-derived portal inflammatory flux; (5) amplification of hepatic inflammation and activation of stellate cells, to drive fibrogenesis; and (6) disrupt further systemic and gut homeostasis via inflammatory mediators. Supporting this model is the fact that it reconciles the parallels we observed in inflammatory programs that are induced in the two organs, the extreme dysbiosis that is present in *Nurr1*-deficient mice, and the results of the AI models being able to classify fibrosis and inflammation simply from structural changes in liver/intestinal samples. It provides firm evidence as to where *Nurr1* is positioned in the bigger picture, between organs and not just tissue/organ/organism, and positions *Nurr1* as a key regulatory node in a multi-organ inflammatory network. We emphasize this cascade is correlative and is a testable hypothesis. Definitive mechanistic attribution requires liver-specific (Alb-Cre), Kupffer cell-specific (LysM-Cre), and intestinal epithelial cell-specific (Vil1-Cre) conditional knockouts, and FMT rescue experiments (35).

This study reconciles contradictory reports in the literature of varying degrees of inflammatory phenotype (23, 36) or lesser effects on metabolic disease (37) when either macrophage- or hepatocyte-specific *Nurr1* knockouts were generated. Our systemic haploinsufficiency has yielded a much more severe outcome due to the combination of losing the regulatory function of *Nurr1* across multiple cell types (hepatocytes, Kupffer cells, intestinal epithelial cells, immune cells) at the same time. It seems likely that the synergy across tissues is driving the extreme phenotype here, that we may not appreciate the full power of transcriptional regulators when studied in a system isolated from their full biology in whole organisms.

The translational implications of this work are substantial. First, the multi-omics signature we define, including high ALT, low *Zo1*, and depletion of *Akkermansia*, identifies candidate pathologies in which *Nurr1* pathway dysfunction may play a role, such as subsets of MASH and IBD, and also points to potential therapeutic opportunities. Secondly, the AI histopathology framework is a roadmap for development of objective (digital) biomarkers for stratifying trials and assessing pathology. Thirdly, our results strongly encourage the exploration of therapeutic *Nurr1* agonists; if activating this pathway can halt this cascade, agonists could have double whammy activity in arresting both hepatic fibrosis and gut inflammation.

Although our model is conceptually coherent, it is not without limitations. We establish correlation; future work utilizing tissue-specific knockout mice or bone marrow chimeras will be required to discern the exact contribution of the hepatocyte, macrophage, and intestinal epithelial *Nurr1* to the overall phenotype. Our dysbiosis analysis was limited to 16S rRNA sequencing; metagenomic and metabolomic profiling would allow for greater functional insights into the degree of change and metabolic output of microbial communities (38). Finally, tests of whether fecal microbiota transplantation from wild-type mice would “rescue” some feedbacks of this phenotype would directly test the causal role of the microbiome.

Also, since these AI models are highly accurate, they were validated on one experimental cohort only and should be tested on external datasets with different staining protocols, scanners, and genetic backgrounds for robust clinical translation (39). Data splitting was performed at image-patch rather than animal level; animal-level partitioning is acknowledged as a limitation and is a priority for future studies. Formal semi-quantitative histological scoring (METAVIR/Ishak for fibrosis; Nancy/Geboes for intestinal inflammation) was not performed and is proposed for future work. histopathological validation using NR4A2-stratified patient biopsies is a key translational future priority. Grad-CAM analysis in Section 3.10 and **Figure 9** now bridges computational and biological understanding by confirming all models attend to histologically meaningful tissue features (40).

## 5 Conclusions

We identified *Nurr1* as a crucial systemic regulator of liver-gut axis homeostasis. In part through the generation and multifaceted analysis of a *Nurr1*-haploinsufficient mouse model, we show that reduced *Nurr1* dosage is sufficient to induce a robust coordinated pathogenic program, consisting of acute hepatocellular injury accompanied by hepatic inflammation and pro-fibrotic reprogramming, together with intestinal epithelial barrier breakdown, mucosal inflammation and a profound loss of commensal microbial community stability.

Our combined CRISPR-Cas9 genome editing, multi-omics profiling, and supervised machine-learning approach ultimately enabled us to define this state as a reproducible and classifiable disease state, and perhaps more interesting, we scaled this molecular signature state to the tissue level by developing and validating deep learning models predicting genotype from standard histopathological slides with near-perfect performance. Classifiers exceeding 99% accuracy on hepatic phenotypes provide quantifiable evidence that *Nurr1* deficient mice induce different tissue-remodeling architectures observed in liver and intestine and that these tissue state patterns can be learned. Together, these findings support a unified pathogenic model where *Nurr1* serves as a gatekeeper, and haploinsufficiency releases a feed-forward loop of intestinal barrier function dysregulation, leading to dysbiosis and culminating in progressive hepatic inflammation and fibrosis. This work experimentally links a specific genetic lesion to a complex and multi-organ phenotype using an integrative methodology, provides a platform for interrogating a new paradigm for *Nurr1* function outside of the brain, disrupts accepted steady state as an archetype for organ communication, and provides a powerful scalable AI framework for the unbiased phenotyping of tissue pathology. Insights from this work place the *Nurr1* pathway as an attractive target for therapeutic purposes and also a source for developing appropriate biomarkers for dysregulated liver-gut diseases.

## Data Availability

The human validation dataset is available in the NCBI Gene Expression Omnibus (GEO), accession GSE164760. All other raw data supporting the conclusions will be made available by the authors without undue reservation.
