# Correction: Nurr1 deficiency orchestrates a coupled liver-gut pathological axis revealed by multi-omics and deep-learning histopathology

**DOI:** 10.3389/fimmu.2026.1917712

**Published:** 2026-07-13

**Authors:** Shah Faisal, Ibad Ullah, Piniel Alphayo Kambey, Abdul Malik, Muhammad Adeel Ejaz, Sajjad Ali Shah, Yin-Xiong Li

**Affiliations:** 1 Center for Cell Lineage Technology and Engineering, Guangdong Provincial Key Laboratory of Biocomputing, Guangzhou Institutes of Biomedicine and Health, Chinese Academy of Sciences, Guangzhou, China; 2 University of Chinese Academy of Sciences, Beijing, China; 3 Faculty of Computing, Riphah International University, Islamabad, Pakistan; 4 Medical Research Institute, Guangdong Provincial People’s Hospital (Guangdong Academy of Medical Sciences), Southern Medical University, Guangzhou, China; 5 China-New Zealand Joint Laboratory on Biomedicine and Health, State Key Laboratory of Respiratory Disease, Guangzhou, China; 6 Institute of Biotechnology and Microbiology, Bacha Khan University, Charsadda, Pakistan

**Keywords:** AI driven phenotyping, convolutional neural network, CRISPR–Cas9 haploinsufficiency, deep learning histopathology, genotype-to-phenotype map, liver–gut axis, multi-omics, *Nurr1* (NR4A2)

There were errors in the author affiliations. A correction has been made to the affiliations that were assigned to several authors and to the affiliations list. The corrected author affiliations appear above.

Author Muhammad Adeel Ejaz was erroneously assigned to affiliation “Medical Research Institute, Guangdong Provincial People’s Hospital (Guangdong Academy of Medical Sciences), Southern Medical University, Guangzhou, China”. This affiliation has now been removed for author Muhammad Adeel Ejaz. Affiliations “Center for Cell Lineage Technology and Engineering, Guangdong Provincial Key Laboratory of Biocomputing, Guangzhou Institutes of Biomedicine and Health, Chinese Academy of Sciences, Guangzhou, China” and “University of Chinese Academy of Sciences, Beijing, China” were omitted for author Muhammad Adeel Ejaz. These affiliations have now been added for author Muhammad Adeel Ejaz.

Author Sajjad Ali Shah was erroneously assigned to affiliation “Guangzhou Institutes of Biomedicine and Health, Chinese Academy of Sciences, Guangzhou, China”. This affiliation has now been removed for author Sajjad Ali Shah. Affiliation “Institute of Biotechnology and Microbiology, Bacha Khan University, Charsadda, Pakistan” was omitted for author Sajjad Ali Shah. This affiliation has now been added for author Sajjad Ali Shah.

Author Yin-Xiong Li was erroneously assigned to affiliation “Institute of Biotechnology and Microbiology, Bacha Khan University, Charsadda, Pakistan”. This affiliation has now been removed for author Yin-Xiong Li. Affiliation “China-New Zealand Joint Laboratory on Biomedicine and Health, State Key Laboratory of Respiratory Disease, Guangzhou, China” was omitted for author Yin-Xiong Li. This affiliation has now been added for author Yin-Xiong Li.

Author Shah Faisal was erroneously assigned to affiliation “China-New Zealand Joint Laboratory on Biomedicine and Health, State Key Laboratory of Respiratory Disease, Guangzhou, China”. This affiliation has now been removed for author Shah Faisal.

Following these changes, the affiliation “Guangzhou Institutes of Biomedicine and Health, Chinese Academy of Sciences, Guangzhou, China”, which appeared as affiliation 6 in the published article, was no longer assigned to any author and has been removed from the article.

The original version of this article has been updated.

